# Generalizing Longitudinal Age Effects on Brain Structure – A Two-Study Comparison Approach

**DOI:** 10.3389/fnhum.2021.635687

**Published:** 2021-04-16

**Authors:** Christiane Jockwitz, Susan Mérillat, Franziskus Liem, Jessica Oschwald, Katrin Amunts, Lutz Jäncke, Svenja Caspers

**Affiliations:** ^1^Institute of Neuroscience and Medicine (INM-1), Research Centre Jülich, Jülich, Germany; ^2^Institute for Anatomy I, Medical Faculty and University Hospital Düsseldorf, Heinrich Heine University, Düsseldorf, Germany; ^3^University Research Priority Program “Dynamics of Healthy Aging”, University of Zurich, Zurich, Switzerland; ^4^C. and O. Vogt Institute for Brain Research, Medical Faculty and University Hospital Düsseldorf, Heinrich Heine University Düsseldorf, Düsseldorf, Germany; ^5^Division of Neuropsychology, University of Zurich, Zurich, Switzerland

**Keywords:** brain structure, aging, cognition, longitudinal change, old age, cortical thickess

## Abstract

Cross-sectional studies indicate that normal aging is accompanied by decreases in brain structure. Longitudinal studies, however, are relatively rare and inconsistent regarding their outcomes. Particularly the heterogeneity of methods, sample characteristics and the high inter-individual variability in older adults prevent the deduction of general trends. Therefore, the current study aimed to compare longitudinal age-related changes in brain structure (measured through cortical thickness) in two large independent samples of healthy older adults (*n* = 161 each); the Longitudinal Healthy Aging Brain (LHAB) database project at the University of Zurich, Switzerland, and 1000BRAINS at the Research Center Juelich, Germany. Annual percentage changes in the two samples revealed stable to slight decreases in cortical thickness over time. After correction for major covariates, i.e., baseline age, sex, education, and image quality, sample differences were only marginally present. Results suggest that general trends across time might be generalizable over independent samples, assuming the same methodology is used, and similar sample characteristics are present.

## Introduction

Normal aging can be accompanied by a decline in cognitive abilities (Hedden and Gabrieli, 2004) and changes in brain structure (Sowell et al., 2003). Both phenomena show high inter-individual variability, especially during later decades of life (Habib et al., 2007; Dickie et al., 2013). Results derived from cross-sectional studies have revealed a negative relationship between age and brain structure across adulthood, with differential effect sizes for specific brain regions (Fjell et al., 2009; Jockwitz et al., 2019), depending on the functional properties of the brain region of interest as well as the brain structure metric investigated (e.g., brain volume-based versus surface-based metrics or cortical thickness versus surface area) (O’Sullivan et al., 2001; Sowell et al., 2003; Salat et al., 2005; Walhovd et al., 2011; Ziegler et al., 2012; Dickie et al., 2013; Hogstrom et al., 2013; Fjell et al., 2014a, b; Liem et al., 2015).

While the associations between brain structure and age are rather heterogenous across studies, we recently showed consistent cross-sectional age associations for two different cohorts when applying the same analysis protocol [e.g., age range, processing of the neuroimaging data (Jockwitz et al., 2019)]. At the same time, cross-sectional studies inherit a potential problem concerning the validity of inferences: Cross-sectional studies assess age-related differences between individuals, which is not comparable to age-related changes within individuals. One important disadvantage of cross-sectional studies concerns interindividual differences that might obscure intraindividual changes of aging (Raz and Lindenberger, 2011).

Longitudinal studies are still relatively rare and inconsistent with respect to their outcomes, preventing the deduction of general trends of age-related changes in brain structure. When comparing cross-sectional and longitudinal research designs, different patterns were shown for structural brain aging (Hedden and Gabrieli, 2004; Pfefferbaum and Sullivan, 2015). Large between-study heterogeneity of designs and methods, differences in sample characteristics and the generally larger inter-individual variability in samples of older adults make it difficult to extract general trends. However, general decreases in brain structure have been reported, although to a lesser degree than those reported in cross-sectional research designs [for a recent review, see Oschwald et al. (2019)].

To extract general age trends for brain structure, comparability between independent study samples is necessary. A few studies have already performed comparability analyses of cross-sectional age-related differences in brain structure metrics (i.e., brain volume or cortical thickness) between different samples, e.g., Fjell et al. (2009); Jockwitz et al. (2019). These studies indicate that general associations between age and brain structure are similar across independent samples, assuming that the same methodology and analysis protocol was used. However, such between study comparisons are lacking for investigations of longitudinal aging trajectories, especially in the older adult population, where inter-individual variability is particularly high. With the growing trend of large imaging consortia, e.g., UK Biobank (Miller et al., 2016), ENIGMA (Thompson et al., 2014), German National Cohort Study [NaKo; Bamberg et al. (2015)], or ADNI [Alzheimer’s Disease Neuroimaging Initiative; Jack et al. (2008)] which aim at pooling datasets from a variety of study centers to increase sample size and statistical power, it will be crucial to establish the validity of age-related changes in brain structure. Therefore, the current study aimed to compare longitudinal age-related changes in brain structure in two large independent samples of healthy older adults: The Longitudinal Healthy Aging Brain (LHAB) database project at the University of Zurich (Switzerland; Zollig et al. (2011)] and 1000BRAINS at the Research Centre Juelich (Germany; Caspers et al. (2014)].

## Materials and Methods

Participants included in the current research project were recruited from two longitudinal studies investigating brain-behavior relationships in older adults located in the larger Zurich area (Switzerland) and in the Ruhr district (Germany).

The first sample comprised the ongoing LHAB database project at the University Research Priority Program (URPP) “Dynamics of Healthy Aging” of the University of Zurich (Zollig et al., 2011). LHAB investigates age-related dynamics of brain-behavior relationships in healthy older adults. A particular focus is placed on assessing and explaining interindividual variability in the observed aging trajectories. For this purpose, a broad spectrum of factors assumed to influence such trajectories (e.g., lifestyle, sleep, and nutrition) is collected. In LHAB, older adults from Zurich and surrounding areas are observed longitudinally with between-measurement intervals of one to 2 years. Inclusion criteria for study participation at baseline were age ≥ 64, right-handedness, fluent German language proficiency, a score of ≥ 26 on the Mini Mental State Examination [MMSE; Folstein et al. (1975)], no self-reported neurological disease of the central nervous system and no contraindications to MRI. The study was approved by the ethical committee of the canton of Zurich. Participation was voluntary and all participants gave written informed consent in accordance with the declaration of Helsinki. The initial sample of LHAB was comprised of 232 participants ranging from 64 to 87 years of age. Data acquisition in the LHAB project started in 2011. Currently the dataset covers an observation period of 7 years.

The second sample comprised 1000BRAINS at the Institute of Neuroscience and Medicine, Research Centre Juelich. 1000BRAINS is a longitudinal population-based study that assesses variability in brain structure and function during aging with respect to various influencing factors (Caspers et al., 2014). The 1000BRAINS sample is drawn from the 10-year follow-up cohort of the Heinz Nixdorf Recall Study, an epidemiological population-based study of risk factors for atherosclerosis, cardiovascular disease, cardiac infarction, and death (Schmermund et al., 2002) and the affiliated MultiGeneration study. In 1000BRAINS, adults aged 55 and older (at baseline) from the Heinz Nixdorf Recall study and their relatives (spouses and offspring; sampled from MultiGeneration study) were recruited, and were examined two times over a period of about 3 to 4 years. In contrast to the LHAB study, inclusion in the study was only dependent on the eligibility requirements for the MR acquisition based on the MR safety guidelines (e.g., stents and heart pacemakers led to exclusion from the study). The study protocol was approved by the University of Duisburg-Essen. Participation was voluntary and all participants gave written informed consent in accordance with the declaration of Helsinki. The initial sample of 1000BRAINS was comprised of 1,315 participants ranging from 18 to 87 years of age.

For the current study, we focused on two time points in both samples (LHAB: baseline and 4-year follow-up; 1000BRAINS: baseline and 3 to 4-years follow-up). Participants with missing values for the brain data were excluded. In order to assure comparability between the two samples, we matched them with respect to baseline age and sex using propensity score matching implemented in R (Stuart et al., 2011).

This resulted in 161 participants for each of the two final samples with the following demographic characteristics: LHAB: mean age = 69.9 ± 4.1; 85 females, mean interval = 4.2 ± 0.1; 1000BRAINS: mean age = 69.2 ± 4.6, 76 females, mean interval = 3.7 ± 0.7. For an overview of demographic variables of the two samples at both timepoints, see Table 1. Education was measured according to the international classification of education (ISCED) and afterward divided into three educational classes: 1. school and/or vocational training, 2. grammar school or vocational baccalaureate, specialized secondary school/diploma, or commercial school degree, and 3. Bachelor, Master, Doctorate or equivalent.

**TABLE 1:** Demographics of the two samples and group comparisons (Independent *T*-test for continuous and Wilxon-Cox test for categorical variables) with corresponding *T*/*W* and *p*-values.

	1000BRAINS	LHAB	*T*/*W* (*P*-Values)
Age (TP1)	69.2 ± 4.6	69.9 ± 4.1	−1.39 (0.166)
Sex	0.53 ± 0.5	0.47 ± 0.5	13685 (0.317)
ISCED 3	2.0 ± 1.0	2.3 ± 0.8	11000 (0.010)
Age (TP2)	72.9 ± 4.7	74 ± 4.1	−2.28 (0.024)
Intervall (TP1 – TP2)	3.7 ± 0.7	4.2 ± 0.1	−8.02 (<0.001)

### Data Acquisition

For LHAB, anatomical T1-weighted images of both timepoints were acquired on a 3.0 T Philips Ingenia scanner (Philips Medical Systems, Best, The Netherlands). T1-weighted structural brain images were measured per visits with: TR = 8.18 ms, TE = 3.8 ms, Flip Angle = 8°, FoV = 240 mm × 240 mm, isotropic voxel size = 1 mm × 1 mm × 1 mm, 160 slices per volume. For 1000BRAINS, anatomical T1-weighted images of both timepoints were acquired on a 3.0 Tesla TIM-Trio MR scanner (Siemens Medical System, Erlangen, Germany). The T1-weighted structural brain images were scanned per visit with: TR = 2.25 s, TE = 3.03 ms, flip angle = 9°, FoV = 256 mm × 256 mm, voxel resolution = 1 mm × 1 mm × 1 mm, 176 slices per volume. In both studies, T1-imaging was part of a larger MR imaging protocol [see Caspers et al. (2014); Zollig et al. (2011)].

### Preprocessing

Anatomical images from both samples were preprocessed using the same automated surface-based processing stream for longitudinal analyses of the FreeSurfer Software package [1000BRAINS: version 6.0.0; LHAB: FreeSurfer BIDS App v6.0.0-2; Gorgolewski et al. (2017)]. A detailed description of this pipeline is provided by Reuter et al. (2012); Dale et al. (1999), Fischl et al. (1999) as well as on http://surfer.nmr.mgh.harvard.edu. In short, first the cross-sectional surface reconstruction pipeline was applied to every subject, which includes (a) the segmentation of the structural brain images into gray matter, white matter, and cerebrospinal fluid, (b) motion correction, (c) intensity normalization, (d) transformation into Talairach space, (e) tessellation of the gray/white matter boundary, and (f) correction of topological defects. The gray/white matter interface was then (g) expanded to create the pial surface (boundary between gray matter and cerebrospinal fluid), which finally consists of about 150,000 vertices per hemisphere with an average surface area of 0.5 mm^2^. Afterwards, each subject was preprocessed using the longitudinal surface reconstruction pipeline (Reuter et al., 2012) in which, based on the results of the cross-sectional preprocessing pipeline, a within-subject anatomical template was built across the two timepoints. Subsequently, cortical thickness was calculated based on the cross-sectional as well as longitudinal information from each subject. This procedure has previously been shown to be more sensitive in calculating surface-based brain metrics, since, due to the common template for the two timepoints, within-subject variability is reduced (Reuter et al., 2012). No manual correction of the reconstructed surfaces (white matter and pial surface) was performed in the two studies.

### Regions of Interest

For the current study, we used the widely used Desikan-Killiany atlas (Desikan et al., 2006) as implemented in FreeSurfer to extract cortical thickness from left and right cortices. Specifically, for each of the 68 regions of interest (ROIs), mean cortical thickness was calculated as the average shortest distance between the white matter surface and the corresponding vertex within the respective ROIs on the pial surface.

### Cognitive Performance

Participants from both LHAB and 1000BRAINS took part in a large neuropsychological assessment consisting of tests in the domains attention, executive functions, working memory, episodic memory and language functions. For comparison between the two samples, the following tasks were chosen: Trail Making Test A: processing speed, B: concept shifting; Morris et al. (1989), LPS50 + subtest three [reasoning; Sturm et al. (1993)] and [Regensburger Wortflüssigkeitstest (RWT), semantic condition (verbal fluency); Aschenbrenner et al. (2000)]. For descriptives of cognitive tasks, see Table 2.

**TABLE 2:** Raw cognitive performance values for TP1 and 2, as well as the APC together with *T* and *p*-values for the APC (Sig. of APC; one sample *T*-test) and *F* and *p*-values for sample homogeneity (Levene’s test).

	1000BRAINS				LHAB				
	Tp1	Tp2	APC	Sig. of APC	Tp1	Tp2	APC	Sig. of APC	Levene’s test
Processing speed	40.22 ± 12.46	41.12 ± 14.12	0.34 ± 7.06	0.61 (0.54)	37.16 ± 12.90	39.37 ± 16.15	1.07 ± 6.88	1.93 (0.056)	0.25 (0.614)
Concept shifting	93.20 ± 41.55	96.87 ± 43.33	0.84 ± 7.98	1.32 (0.188)	86.69 ± 33.86	94.22 ± 39.77	2.04 ± 6.83	3.63 (<0.001)	2.40 (0.122)
Verbal fluency	23.96 ± 6.67	22.81 ± 6.73	−1.31 ± 5.76	−2.81 (0.006)	26.06 ± 6.46	25.98 ± 5.83	0.17 ± 4.41	0.47 (0.633)	9.59 (0.002)
Reasoning	20.99 ± 4.65	20.56 ± 5.42	−0.13 ± 5.14	−0.31 (0.757)	24.02 ± 4.45	26.48 ± 4.75	2.35 ± 3.70	7.99 (<0.001)	10.66 (0.001)

### Statistical Analysis

The purpose of the current research project was to compare intra-individual changes in brain structure (cortical thickness) across the ROIs of two independent population-based cohort studies. We calculated annual percentage changes to estimate yearly changes in cortical thickness and cognitive performance. Annual percentage changes were calculated as the following: [(Value at last measurement occasion in the study/Value at baseline)^1/(total years in study)^−1] × 100. Positive values represent increases and negative values represent decreases. We next identified outliers for all annual percentage changes (mean annual percentage change ± 3 SD) and excluded those values that deviated more than 3 SD from the mean.

To examine whether the two samples showed similar changes in cortical thickness over time, we first used a one sample *t*-test to estimate general changes in cortical thickness for the two groups separately. To investigate whether the two samples differed concerning their variances, we conducted Levene’s test for sample homogeneity. Finally, between sample differences in cortical thickness annual percentage changes were assessed using a General Linear Model (GLM) with cortical thickness as the dependent variable and sample and sex as fixed factors. Baseline age (TP1), education, and Euler number were included as covariates of non-interest. Euler number represents a marker of image quality that summarizes the topological complexity of the reconstructed cortical surface (Rosen et al., 2018).

Subsequently, we assessed the cortical thickness annual percentage changes with the mentioned covariates (baseline age, sex, education, and Euler number) separately for the two samples to examine whether changes in cortical thickness would be driven by one sample. Finally, we additionally assessed the relation between annual percentage changes of cortical thickness and cognitive performance for the two samples separately.

## Results

When matching the two samples for baseline age and sex, the two samples did not differ in the respective variables (baseline age: *T* = −1.39, *p* = 0.166; and sex: *W* = 13,685, *p* = 0.317). However, we found significant differences in terms of education (*W* = 11,000, *p* = 0.01), with participants included in LHAB generally showing a higher formal education as compared to participants included in 1000BRAINS. Furthermore, the time intervals between the two measurements differed, with a longer interval between measurements in the LHAB project (1000BRAINS: 3.7 ± 0.7 years; LHAB: 4.2 ± 0.1 years; *T* = −8.02; *p* < 0.001; for group differences, see Table 1). To address this difference in time intervals we calculated annual percentage changes of cortical thickness. Table 3 includes cortical thickness values for the two hemispheres at both timepoints as well as the annual percentage change in cortical thickness for the two samples separately (for all ROIs see Supplementary Table 1).

**TABLE 3:** Cortical thickness values for TP1 and 2, as well as the annual percentage change (APC) together with *T* and *p*-values for the APC (Sig. of APC; one sample *T*-test) and *F* and *p*-values for sample homogeneity (Levene’s test).

	1000BRAINS				LHAB				
	Tp1	Tp2	APC	Sig. of APC	Tp1	Tp2	APC	Sig. of APC	Levene’s test
Mean CT left	2.46 ± 0.09	2.45 ± 0.09	−0.15 ± 0.45	−4.17 (<0.001)	2.4 ± 0.08	2.37 ± 0.09	−0.29 ± 0.45	−8.21 (<0.001)	0.17 (0.677)
Mean CT right	2.46 ± 0.09	2.45 ± 0.10	−0.14 ± 0.40	−4.49 (<0.001)	2.41 ± 0.08	2.38 ± 0.09	−0.3 ± 0.42	−9.07 (<0.001)	0.19 (0.664)

### Cortical Thickness

**FIGURE 1:**
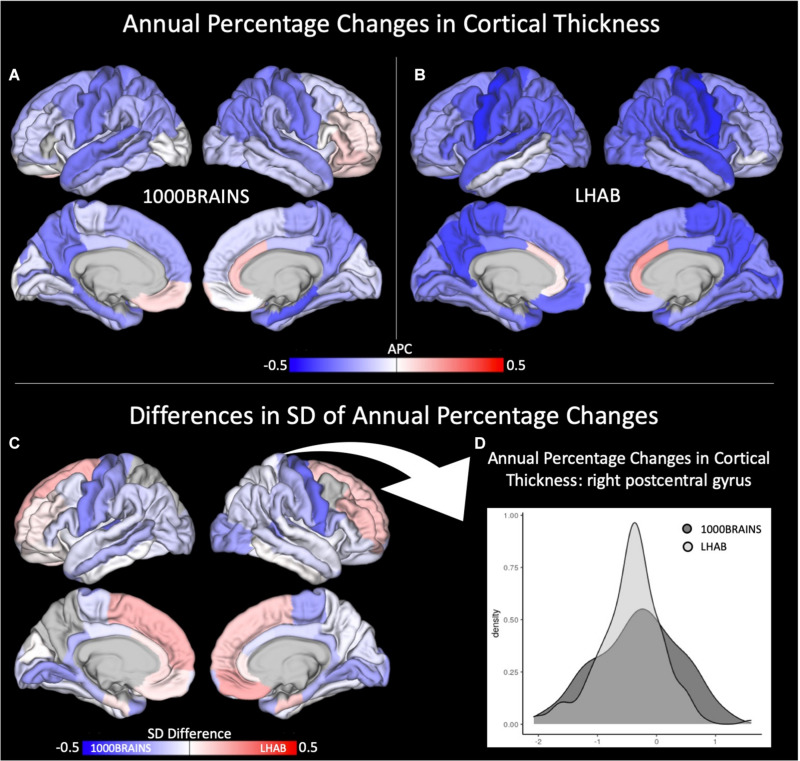
Annual percentage changes (APC) in cortical thickness for **(A)** 1000BRAINS and **(B)** LHAB. Differences in SD between the two samples is shown in **(C)** together with a corresponding density plot **(D)** showing the variance in cortical thickness for 1000BRAINS and LHAB within the postcentral gyrus.

With respect to cortical thickness, the LHAB sample showed slightly stronger annual percentage changes (i.e., decreases) in cortical thickness over time as compared to 1000BRAINS (see Figures 1A,B). On the other hand, we found 1000BRAINS to generally show more variance between participants regarding the annual percentage change in most of the ROIs (for Levene’s test, Supplementary Table 1), although variances in mean CT did not differ significantly between the two samples (see Table 3). Figure 1C shows difference maps in terms of standard deviations of the annual percentage changes. For example, one of the most significant differences in standard deviations is observed in the right postcentral gyrus (see Figure 1C for a density plot; 1000BRAINS: SD = 0.7, LHAB: SD = 0.5; Levene’s test: *F* = 14.64, *p* < 0.001).

Next, we again used GLMs to examine sample differences in annual percentage changes in cortical thickness with age, sex, education and Euler number as covariates (for all significant influences, see Table 4 and Supplementary Table 2). Overall, after correcting for the different covariates and for multiple comparisons, only very few sample differences in terms of annual percentage change were present, i.e., inferior frontal gyrus pars triangularis (lh: *F* = 13.67, rh: *F* = 16.54) and inferior frontal gyrus pars opercularis (rh: *F* = 21.43) and transverse temporal gyrus (rh: *F* = 20.47).

**TABLE 4:** *F* and *p*-values derived from general linear models assessing annual percentage changes in cortical thickness in relation to sample, age, sex, education, and data quality (Euler number).

	Intercept	Age (TP1)	Sex	Education	Euler	Sample
Mean CT left	1.83 (0.177)	2.41 (0.121)	0.00 (0.966)	0.10 (0.756)	0.94 (0.334)	7.5 (0.007)
Mean CT right	4.35 (0.038)	4.95 (0.027)	0.44 (0.508)	0.95 (0.331)	0.32 (0.572)	8.85 (0.003)

**FIGURE 2:**
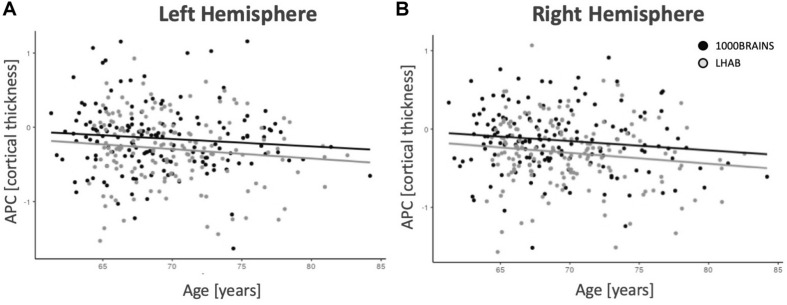
Mean thickness annual percentage changes for the left **(A)** and right **(B)** hemispheres. With increasing age, there are slightly decreasing annual percentage changes for both samples.

In addition, after correcting for the above-mentioned variables, only a few regions showed significant intercepts (i.e., main effects of time), age effects or relations to sex, education or the Euler number (almost no effects did survive correction for multiple comparisons). Figure 2 shows age-related annual percentage changes in cortical thickness for left and right hemispheres. As one can see in the two plots, the annual percentage change was not significantly related to baseline age for the left hemisphere (*F* = 2.41; *p* = 0.121) but was at trend level for the right hemisphere (*F* = 4.95; *p* = 0.027). The plots also show that the relationship between age and annual percentage change follows a linear, rather than a non-linear trend.

**FIGURE 3:**
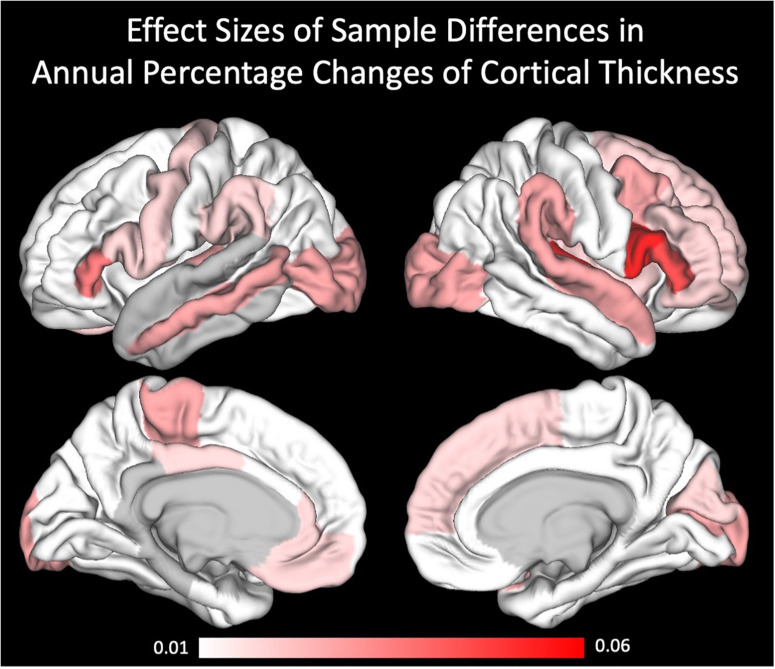
Effect sizes of sample differences using partial eta square.

For a better understanding of the regional specificity of sample differences in the cortical thickness annual percentage changes, we projected the effect sizes (partial eta squared) of the sample differences onto the brains surface (Figure 3). Effect sizes ranged from 0 to 0.06, being interpreted as small to medium effects. Regarding the covariates, we only found sporadic effects on cortical thickness annual percentage change. After correcting for these subtle, mostly non-significant influences, and even the intercepts (i.e., main effects of annual percentage change) became non-significant. To verify that these influences were not driven by only one of the two samples, we further calculated the GLMs for the two samples separately (see Supplementary Table 3).

Finally, we assessed the relation between annual percentage changes of cortical thickness and cognitive performance for the two samples separately, which, after correcting for multiple comparisons, revealed non-significant results (see Tables 2, 5 and Supplementary Table 4).

**TABLE 5:** *F* and *p*-values derived from general linear models assessing the relation between annual percentage changes in cortical thickness with annual percentage changes in cognitive performance, calculated separately for the two samples, corrected for age, sex, education, and data quality (Euler number).

	Processing speed		Concept shifting		Verbal fluency		Reasoning	
	1000BRAINS	LHAB	1000BRAINS	LHAB	1000BRAINS	LHAB	1000BRAINS	LHAB
Mean CT left	0.21 (0.651)	5.45 (0.021)	1.27 (0.263)	0.31 (0.581)	0.26 (0.609)	0.00 (0.997)	2.40 (0.124)	1.03 (0.311)
Mean CT right	1.55 (0.215)	2.63 (0.107)	0.03 (0.864)	0.00 (0.971)	0.45 (0.505)	0.41 (0.522)	0.49 (0.484)	3.50 (0.063)

## Discussion

Generalizability and replicability of age effects on brain and behavior are vital requirements to understand major aging mechanisms in our older adult population. The complexity of the aging process, in which the effect of single contributing factors, i.e., lifestyle or genetics, is assumed to be highly individual and rather small. To unravel even subtle brain-behavior relationships during aging (Button et al., 2013; Wiseman et al., 2019) there is an upcoming trend of data pooling approaches to increase statistical power. However, data pooling procedures, particularly in imaging consortia, require proof of generalizability of observed age-related brain changes. The present study set out to meet this need and assessed age-related changes in brain structure (measured by global and regional cortical thickness) in two closely matched samples of older adults over an average time period of three to four years. Despite significant differences in demographics between the two independent samples, we observed highly similar patterns of age-related changes in brain structure, when using the same methodology and analysis.

Cross-sectional age-related atrophy patterns have been reported by many previous studies (Walhovd et al., 2011; Storsve et al., 2014; Jancke et al., 2015). From those studies we would have expected to see a pattern of small but consistent cortical thickness decline in our two studies.

Overall, this decrease was found for both studies (cf. Figure 1) with participants included in the LHAB study showing a slightly more pronounced decline in cortical thickness. Highest annual percentage changes were found for pre- and postcentral gyri together with medial and lateral temporal and parietal brain regions in both samples. In turn, the anterior cingulate cortex showed slight increases in cortical thickness over time. Importantly, the results are in line with previous longitudinal studies on cortical thickness investigating the whole adult lifespan (Storsve et al., 2014). Further, sample inhomogeneity testing revealed a higher between-subject variance for 1000BRAINS as compared to the LHAB study.

When adjusting the longitudinal effects of time for sex, education, baseline age and data quality (Euler number), only sporadic brain areas exhibited significant sample effects in annual percentage changes, i.e., left and right inferior frontal gyrus, pars triangularis, right inferior frontal gyrus pars opercularis and the right transverse temporal gyrus. Here, participants included in the LHAB study showed a more pronounced decrease over time. Based on sample characteristics, e.g., higher education in the LHAB sample, one would expect 1000BRAINS to show a more pronounced cortical thinning. However, especially for the inferior frontal gyrus (i.e., Broca’s region involved in language functions), it has been shown that a higher brain reserve, in terms of higher gray matter volume, may diminish during the aging process, i.e., at older ages (Heim et al., 2019). If this holds true, then it might be the case that participants of the two samples assimilate during older ages in terms of brain structure. However, further research is necessary to unravel this complex relationship of age and brain structure.

Thus, the analysis of cortical thickness in two samples of healthy older adults revealed only marginal changes over time and only minimal sample differences. We are aware that our models include more covariate variables (age, sex, education, and data quality) than previous studies [e.g., Walhovd et al. (2011); Storsve et al. (2014); Thambisetty et al. (2010)]. We deliberately decided to include this set of variables since we know from previous research that cross-sectionally, the factors age, sex, education and data quality have an impact on brain structure (Sowell et al., 2003; Jancke et al., 2015; Jockwitz et al., 2019). Interestingly, when examining “raw annual percentage changes,” these changes were partly in accordance with previous studies investigating changes in cortical thickness over time (Walhovd et al., 2011; Storsve et al., 2014). For example, Storsve found a mean annual percentage change of −0.35 in a sample ranging from 23 – 87 years and Fjell et al. (2014b) reported a mean annual percentage change of −0.59 in a sample of older adults. While we found a mean annual percentage change of −0.29 for the LHAB study, in 1000BRAINS this was slightly less pronounced, i.e., −0.15. In addition, we showed that the investigated covariates, i.e., baseline age, sex, education, and image quality, might be important in the investigation of longitudinal changes of brain structure. As an example, we found slightly negative relationships between baseline age and annual percentage changes in cortical thickness for the right hemisphere, which supports previous results (e.g., Fjell et al., 2009).

Finally, it has to be mentioned that neither of the two studies showed significant relations between annual percentage changes in cortical thickness and cognitive performance (i.e., processing speed, concept shifting, verbal fluency, and reasoning). First, these results complement previous results of our research group. In this cross-sectional study, no relation between cortical thickness and cognitive performance could be established in neither of the two study samples (Jockwitz et al., 2019). Likewise, other studies also revealed no associations between cognitive performance and particularly cortical thickness (in contrast to, e.g., brain volume [Cox et al., 2019], or white matter [Ziegler et al., 2012]). Furthermore, research regarding changes in both, brain structure and cognitive performance is quite heterogeneous. In the literature review of Oschwald et al. (2019) half of the studies revealed no association between changes in brain structure and cognitive performance, which fits to the current observation. In turn, those studies showing a significant association between changes in particularly cortical thickness and cognitive performance, differed from the current study. First, other cognitive functions were investigated, such as episodic memory or composite scores of executive functions (Fjell et al., 2014b; Möller et al., 2016; Sala-Llonch et al., 2017) and second, the above-mentioned studies included less or no covariates. Thus, when correcting for major confounding effects, cortical thickness changes were not related to cognitive performance changes over time. This is also well in line with the idea that in healthy older adults, correlations between changes in brain structure and simultaneous changes in cognitive performance are expectedly small and accompanied by high amounts of variability due to potential compensation mechanisms (Oschwald et al., 2019).

### Methodological Considerations

The current study assessing longitudinal changes in brain structure has several advantages as well as limitations that we would like to address. With respect to the brain metric used in the current study, we chose cortical thickness, since it represents a prominent brain metric that seems to be sensitive to the aging process. However, it should also be mentioned that other metrics might be useful when comparing effects of aging, i.e., brain volume or gray matter density (Jäncke et al., 2019). Also, future studies may adopt Deformation-Field Morphometry methods, such as Tensor-based morphometry (TBM), in order to compute longitudinal change in structural MRI data (Hua et al., 2008). Furthermore, with regard to the atlas used in the current study, i.e., Desikan-Killiany atlas, it needs to be stressed that other atlases might be more sensitive to functionally dependent changes in brain structure, such as the cytoarchitectonic Juelich Brain Atlas (Amunts et al., 2020) or functionally derived brain parcellations (Schaefer et al., 2018). In addition, future studies should also investigate longitudinal changes in brain structure and function with samples that are matched not only for age and gender, but also education or cognitive abilities. In the current study, we showed that covariates, such as age and education might explain small parts of the changes seen over time. Future studies should elaborate on these influencing factors to explore intra-individual aging processes.

## Conclusion

Taken together, the current study showed that age-related changes in cortical thickness are relatively small, when adjusting for the most common influencing factors. This effect was seen in both independent studies, suggesting that general patterns of longitudinal changes in brain structure may be generalizable if the same methods are used and similar study populations with similar age and sex distributions are selected. However, fine-grained change patterns differ and the question whether results can be generalized over different samples cannot easily be answered because of the between-study differences regarding demographics (e.g., age ranges and education) or methodology (e.g., time intervals, different brain metrics, and such as brain volume versus cortical thickness). Furthermore, differences in covariates often hamper the extraction of generalizable age trends in different samples. With our study, we contribute to the field by showing that patterns of age-related changes in brain structure in two independent cohorts of older adults are highly similar when using the same methodological approach.

## Data Availability Statement

The datasets presented in this article are not readily available because the used consent does not allow for the public sharing of the data. Requests to access the datasets should be directed to LJ, lutz.jaencke@uzh.ch (LHAB) and SC, s.caspers@fz-juelich.de (1000BRAINS).

## Ethics Statement

The studies involving human participants were reviewed and approved by the Ethical Committee of the Canton of Zurich, Switzerland (LHAB) and the University of Duisburg-Essen, Germany (1000BRAINS). The participants provided their written informed consent to participate in this study.

## Author Contributions

SM and LJ contributed to the design, set-up, maintenance, and support of the LHAB project. SC and KA contributed to the design, set-up, maintenance, and support of the 1000BRAINS study. FL and CJ performed processing of the longitudinal neuroimaging data and wrote the first draft of the manuscript. CJ and JO performed the statistical analysis. LJ and SC supervised the project. All authors discussed the results, contributed to manuscript revision, and read and approved the submitted version.

## Conflict of Interest

The authors declare that the research was conducted in the absence of any commercial or financial relationships that could be construed as a potential conflict of interest.
