# Supplementary material for: Generalizing Longitudinal Age Effects on Brain Structure – A Two-Study Comparison Approach
Source: Front Hum Neurosci. 2021 Apr 16;15:635687. doi: 10.3389/fnhum.2021.635687 (PMC8085300; doi:10.3389/fnhum.2021.635687)
Supplement: Supplementary file 1 [file Table_1.docx]

**Generalizing longitudinal age effects on brain structure**

**– a two-study comparison approach**

***SUPPLEMENT***

**C. Jockwitz^1,2^, S. Mérillat^3^, F. Liem^3^, J. Oschwald^3^, K. Amunts^1,4^,**

**L. Jäncke^3,5^*, S. Caspers^1,2^***

*^1^ Institute of Neuroscience and Medicine (INM-1), Research Centre Jülich, Jülich, Germany*

*^2^* *Institute for Anatomy I, Medical Faculty & University Hospital Düsseldorf, Heinrich Heine University Düsseldorf, Düsseldorf, Germany*

*^3^University Research Priority Program “Dynamics of Healthy Aging”, University of Zurich, Zurich, Switzerland*

*^4^C. & O. Vogt Institute for Brain Research, Medical Faculty & University Hospital Düsseldorf, Heinrich Heine University Düsseldorf, Düsseldorf, Germany*

*^5^Division of Neuropsychology, University of Zurich, Zurich, Switzerland*

*****these authors contributed equally

Table S1: Cortical thickness values for TP1 and TP2, as well as the annual percentage change (APC) together with T and p values for the APC (Sign. APC; One sample T-Test) and F and p values for sample homogeneity (Levene’s Test).

|  |  | 1000B | | | | LHAB | | | |  |
| --- | --- | --- | --- | --- | --- | --- | --- | --- | --- | --- |
|  | **Region** | **Tp1** | **Tp2** | **APC** | **Sign. APC** | **Tp1** | **Tp2** | **APC** | **Sign. APC** | **Levene‘s Test** |
| left | bankssts | 2.43 ± 0.14 | 2.42 ± 0.15 | -0.14 ± 0.72 | -2.42 (0.017) | 2.43 ± 0.12 | 2.41 ± 0.13 | -0.26 ± 0.57 | -5.72 (<.001) | 6.71 (0.01) |
|  | caudalanterior cingulate | 2.72 ± 0.26 | 2.72 ± 0.27 | 0.00 ± 0.78 | -0.08 (0.936) | 2.60 ± 0.24 | 2.61 ± 0.25 | 0.09 ± 0.84 | 1.36 (0.176) | 0.05 (0.825) |
|  | caudalmiddlefrontal | 2.59 ± 0.14 | 2.56 ± 0.15 | -0.24 ± 0.76 | -4.03 (<.001) | 2.45 ± 0.12 | 2.41 ± 0.12 | -0.35 ± 0.70 | -6.29 (<.001) | 0.23 (0.635) |
|  | cuneus | 1.84 ± 0.12 | 1.83 ± 0.12 | -0.07 ± 0.71 | -1.27 (0.207) | 1.83 ± 0.12 | 1.81 ± 0.11 | -0.25 ± 0.72 | -4.37 (<.001) | 0.03 (0.862) |
|  | entorhinal | 3.49 ± 0.32 | 3.45 ± 0.37 | -0.25 ± 0.97 | -3.26 (0.001) | 3.50 ± 0.28 | 3.45 ± 0.32 | -0.28 ± 1.03 | -3.42 (<.001) | 0.35 (0.557) |
|  | frontalpole | 2.74 ± 0.25 | 2.74 ± 0.26 | 0.03 ± 0.99 | 0.35 (0.731) | 2.58 ± 0.20 | 2.56 ± 0.21 | -0.12 ± 0.93 | -1.60 (0.112) | 0.00  (0.989) |
|  | fusiform | 2.73 ± 0.12 | 2.72 ± 0.12 | -0.15 ± 0.59 | -3.30 (0.001) | 2.70 ± 0.12 | 2.67 ± 0.13 | -0.22 ± 0.54 | -5.16 (<.001) | 2.08 (0.15) |
|  | inferiorparietal | 2.45 ± 0.11 | 2.42 ± 0.13 | -0.22 ± 0.67 | -4.13 (<.001) | 2.34 ± 0.10 | 2.31 ± 0.11 | -0.34 ± 0.59 | -7.31 (<.001) | 1.66 (0.199) |
|  | inferiortemporal | 2.73 ± 0.12 | 2.72 ± 0.13 | -0.11 ± 0.61 | -2.37 (0.019) | 2.68 ± 0.11 | 2.67 ± 0.11 | -0.13 ± 0.60 | -2.66 (0.009) | 0.13 (0.716) |
|  | insula | 2.94 ± 0.16 | 2.93 ± 0.16 | -0.09 ± 0.70 | -1.72 (0.087) | 2.90 ± 0.18 | 2.86 ± 0.18 | -0.28 ± 0.67 | -5.29 (<.001) | 0.00  (0.966) |
|  | isthmuscingulate | 2.28 ± 0.18 | 2.26 ± 0.19 | -0.32 ± 0.91 | -4.40 (<.001) | 2.32 ± 0.18 | 2.28 ± 0.18 | -0.41 ± 0.74 | -7.00 (<.001) | 6.54 (0.011) |
|  | lateraloccipital | 2.24 ± 0.14 | 2.23 ± 0.12 | -0.01 ± 0.68 | -0.11 (0.912) | 2.17 ± 0.12 | 2.14 ± 0.12 | -0.26 ± 0.58 | -5.80 (<.001) | 3.21 (0.074) |
|  | lateralorbitofrontal | 2.64 ± 0.12 | 2.64 ± 0.12 | -0.01 ± 0.64 | -0.22 (0.83) | 2.61 ± 0.14 | 2.60 ± 0.15 | -0.14 ± 0.66 | -2.62 (0.01) | 0.85 (0.356) |
|  | lingual | 1.94 ± 0.11 | 1.93 ± 0.12 | -0.06 ± 0.86 | -0.84 (0.401) | 1.98 ± 0.12 | 1.95 ± 0.11 | -0.26 ± 0.64 | -5.21 (<.001) | 13.34 (<.001) |
|  | medialorbitofrontal | 2.39 ± 0.14 | 2.40 ± 0.14 | 0.09 ± 0.87 | 1.28 (0.201) | 2.28 ± 0.13 | 2.25 ± 0.13 | -0.33 ± 0.93 | -4.47 (<.001) | 0.73 (0.393) |
|  | middletemporal | 2.79 ± 0.14 | 2.76 ± 0.15 | -0.22 ± 0.63 | -4.35 (<.001) | 2.71 ± 0.12 | 2.70 ± 0.12 | -0.05 ± 0.47 | -1.34 (0.183) | 10.5 (0.001) |
|  | paracentral | 2.42 ± 0.15 | 2.41 ± 0.16 | -0.09 ± 0.91 | -1.18 (0.241) | 2.35 ± 0.14 | 2.32 ± 0.15 | -0.38 ± 0.83 | -5.83 (<.001) | 1.67 (0.197) |
|  | parahippocampal | 2.75 ± 0.27 | 2.72 ± 0.29 | -0.29 ± 0.81 | -4.42 (<.001) | 2.88 ± 0.27 | 2.86 ± 0.29 | -0.20 ± 0.66 | -3.77 (<.001) | 6.49 (0.011) |
|  | parsopercularis | 2.56 ± 0.13 | 2.54 ± 0.13 | -0.09 ± 0.70 | -1.62 (0.107) | 2.49 ± 0.10 | 2.46 ± 0.11 | -0.30 ± 0.60 | -6.32 (<.001) | 2.93 (0.088) |
|  | parsorbitalis | 2.69 ± 0.17 | 2.67 ± 0.18 | -0.14 ± 0.83 | -2.13 (0.034) | 2.61 ± 0.16 | 2.59 ± 0.18 | -0.20 ± 0.85 | -3.00 (0.003) | 0.03 (0.859) |
|  | parstriangularis | 2.41 ± 0.14 | 2.40 ± 0.14 | 0.00 ± 0.76 | -0.02 (0.986) | 2.36 ± 0.12 | 2.32 ± 0.12 | -0.34 ± 0.67 | -6.46 (<.001) | 2.82 (0.094) |
|  | pericalcarine | 1.61 ± 0.12 | 1.59 ± 0.12 | -0.22 ± 1.04 | -2.63 (0.009) | 1.60 ± 0.13 | 1.57 ± 0.12 | -0.37 ± 0.95 | -4.94 (<.001) | 0.75 (0.387) |
|  | postcentral | 2.08 ± 0.11 | 2.06 ± 0.12 | -0.28 ± 0.77 | -4.57 (<.001) | 2.01 ± 0.10 | 1.98 ± 0.10 | -0.39 ± 0.57 | -8.76 (<.001) | 7.56 (0.006) |
|  | posteriorcingulate | 2.49 ± 0.15 | 2.48 ± 0.16 | -0.13 ± 0.76 | -2.18 (0.031) | 2.48 ± 0.16 | 2.45 ± 0.15 | -0.29 ± 0.69 | -5.19 (<.001) | 2.20 (0.139) |
|  | precentral | 2.53 ± 0.15 | 2.51 ± 0.16 | -0.31 ± 0.97 | -3.96 (<.001) | 2.50 ± 0.12 | 2.45 ± 0.14 | -0.48 ± 0.70 | -8.79 (<.001) | 7.73 (0.006) |
|  | precuneus | 2.38 ± 0.12 | 2.35 ± 0.13 | -0.29 ± 0.59 | -6.20 (<.001) | 2.32 ± 0.11 | 2.28 ± 0.13 | -0.42 ± 0.59 | -9.09 (<.001) | 0.41 (0.522) |
|  | rostralanteriorcingulate | 2.75 ± 0.20 | 2.75 ± 0.20 | 0.00 ± 0.67 | -0.04 (0.968) | 2.66 ± 0.21 | 2.66 ± 0.23 | 0.05 ± 0.75 | 0.83 (0.41) | 0.79 (0.376) |
|  | rostralmiddlefrontal | 2.36 ± 0.12 | 2.35 ± 0.13 | -0.10 ± 0.65 | -1.88 (0.062) | 2.26 ± 0.09 | 2.25 ± 0.11 | -0.21 ± 0.70 | -3.71 (<.001) | 0.08 (0.774) |
|  | superiorfrontal | 2.69 ± 0.14 | 2.67 ± 0.14 | -0.20 ± 0.49 | -5.05 (<.001) | 2.55 ± 0.11 | 2.52 ± 0.12 | -0.25 ± 0.66 | -4.75 (<.001) | 10.72 (0.001) |
|  | superiorparietal | 2.24 ± 0.13 | 2.21 ± 0.15 | -0.25 ± 0.60 | -5.20 (<.001) | 2.12 ± 0.11 | 2.09 ± 0.12 | -0.30 ± 0.60 | -6.42 (<.001) | 0.00 (0.994) |
|  | superiortemporal | 2.70 ± 0.13 | 2.67 ± 0.14 | -0.30 ± 0.62 | -6.25 (<.001) | 2.66 ± 0.13 | 2.62 ± 0.14 | -0.37 ± 0.56 | -8.30 (<.001) | 1.88 (0.172) |
|  | supramarginal | 2.52 ± 0.11 | 2.50 ± 0.12 | -0.17 ± 0.61 | -3.57 (<.001) | 2.44 ± 0.10 | 2.40 ± 0.11 | -0.33 ± 0.51 | -8.08 (<.001) | 6.02 (0.015) |
|  | temporalpole | 3.70 ± 0.29 | 3.66 ± 0.32 | -0.19 ± 0.99 | -2.38 (0.019) | 3.64 ± 0.28 | 3.59 ± 0.32 | -0.36 ± 0.79 | -5.67 (<.001) | 7.47 (0.007) |
|  | transversetemporal | 2.27 ± 0.20 | 2.26 ± 0.20 | -0.16 ± 1.28 | -1.53 (0.128) | 2.33 ± 0.20 | 2.26 ± 0.20 | -0.66 ± 0.95 | -8.76 (<.001) | 13.49 (<.001) |
| right | bankssts | 2.53 ± 0.15 | 2.51 ± 0.15 | -0.24 ± 0.82 | -3.76 (<.001) | 2.52 ± 0.14 | 2.49 ± 0.14 | -0.34 ± 0.56 | -7.71 (<.001) | 13.30 (<.001) |
|  | caudalanteriorcingulate | 2.53 ± 0.22 | 2.55 ± 0.22 | 0.14 ± 0.81 | 2.13 (0.035) | 2.46 ± 0.21 | 2.48 ± 0.22 | 0.23 ± 0.73 | 3.92 (<.001) | 1.12 (0.29) |
|  | caudalmiddlefrontal | 2.55 ± 0.14 | 2.54 ± 0.15 | -0.12 ± 0.69 | -2.18 (0.031) | 2.45 ± 0.11 | 2.42 ± 0.12 | -0.35 ± 0.69 | -6.37 (<.001) | 0.00 (0.981) |
|  | cuneus | 1.89 ± 0.13 | 1.89 ± 0.13 | -0.06 ± 0.74 | -0.99 (0.324) | 1.84 ± 0.11 | 1.82 ± 0.11 | -0.26 ± 0.67 | -5.03 (<.001) | 1.44 (0.231) |
|  | entorhinal | 3.59 ± 0.33 | 3.54 ± 0.36 | -0.37 ± 0.92 | -5.14 (<.001) | 3.57 ± 0.29 | 3.54 ± 0.34 | -0.21 ± 1.04 | -2.49 (0.014) | 0.26 (0.612) |
|  | frontalpole | 2.68 ± 0.24 | 2.69 ± 0.25 | 0.09 ± 1.05 | 1.07 (0.285) | 2.58 ± 0.19 | 2.57 ± 0.20 | -0.10 ± 0.92 | -1.41 (0.161) | 3.08 (0.08) |
|  | fusiform | 2.74 ± 0.13 | 2.72 ± 0.14 | -0.18 ± 0.63 | -3.58 (<.001) | 2.71 ± 0.11 | 2.68 ± 0.13 | -0.29 ± 0.58 | -6.21 (<.001) | 0.95 (0.331) |
|  | inferiorparietal | 2.47 ± 0.13 | 2.45 ± 0.13 | -0.28 ± 0.60 | -5.86 (<.001) | 2.38 ± 0.11 | 2.35 ± 0.12 | -0.36 ± 0.52 | -8.94 (<.001) | 5.17 (0.024) |
|  | inferiortemporal | 2.75 ± 0.13 | 2.73 ± 0.15 | -0.10 ± 0.50 | -2.50 (0.013) | 2.73 ± 0.13 | 2.71 ± 0.13 | -0.19 ± 0.51 | -4.83 (<.001) | 0.04 (0.847) |
|  | insula | 2.92 ± 0.17 | 2.90 ± 0.17 | -0.07 ± 0.70 | -1.31 (0.193) | 2.88 ± 0.17 | 2.83 ± 0.19 | -0.34 ± 0.67 | -6.55 (<.001) | 0.67 (0.415) |
|  | isthmuscingulate | 2.32 ± 0.19 | 2.30 ± 0.19 | -0.23 ± 0.90 | -3.25 (0.001) | 2.34 ± 0.21 | 2.30 ± 0.20 | -0.38 ± 0.75 | -6.36 (<.001) | 6.36 (0.012) |
|  | lateraloccipital | 2.28 ± 0.13 | 2.27 ± 0.13 | -0.12 ± 0.75 | -2.01 (0.046) | 2.23 ± 0.12 | 2.20 ± 0.13 | -0.31 ± 0.54 | -7.34 (<.001) | 21.59 (<.001) |
|  | lateralorbitofrontal | 2.59 ± 0.13 | 2.60 ± 0.14 | 0.08 ± 0.73 | 1.32 (0.19) | 2.54 ± 0.13 | 2.53 ± 0.12 | -0.14 ± 0.67 | -2.71 (0.007) | 0.54 (0.462) |
|  | lingual | 2.01 ± 0.12 | 2.01 ± 0.11 | -0.11 ± 0.84 | -1.63 (0.106) | 2.01 ± 0.11 | 1.98 ± 0.11 | -0.28 ± 0.64 | -5.62 (<.001) | 8.74 (0.003) |
|  | medialorbitofrontal | 2.43 ± 0.13 | 2.43 ± 0.13 | -0.02 ± 0.61 | -0.39 (0.698) | 2.33 ± 0.14 | 2.31 ± 0.14 | -0.16 ± 0.80 | -2.57 (0.011) | 10.32 (0.001) |
|  | middletemporal | 2.82 ± 0.13 | 2.80 ± 0.15 | -0.11 ± 0.56 | -2.38 (0.018) | 2.79 ± 0.12 | 2.77 ± 0.13 | -0.16 ± 0.48 | -4.31 (<.001) | 2.62 (0.107) |
|  | paracentral | 2.48 ± 0.15 | 2.46 ± 0.16 | -0.20 ± 0.92 | -2.72 (0.007) | 2.34 ± 0.13 | 2.30± 0.14 | -0.39 ± 0.72 | -6.71 (<.001) | 2.92 (0.089) |
|  | parahippocampal | 2.69 ± 0.24 | 2.65 ± 0.25 | -0.39 ± 0.82 | -6.01 (<.001) | 2.80 ± 0.23 | 2.77 ± 0.25 | -0.26 ± 0.66 | -5.00 (<.001) | 7.47 (0.007) |
|  | parsopercularis | 2.55 ± 0.13 | 2.55 ± 0.14 | 0.01 ± 0.69 | 0.09 (0.926) | 2.51 ± 0.13 | 2.48 ± 0.14 | -0.28 ± 0.52 | -6.73 (<.001) | 4.02 (0.046) |
|  | parsorbitalis | 2.65 ± 0.18 | 2.66 ± 0.19 | 0.11 ± 0.76 | 1.88 (0.062) | 2.58 ± 0.19 | 2.57 ± 0.19 | -0.04 ± 0.70 | -0.80 (0.427) | 0.57 (0.452) |
|  | parstriangularis | 2.40 ± 0.13 | 2.41 ± 0.14 | 0.10 ± 0.65 | 2.00 (0.047) | 2.37 ± 0.11 | 2.35 ± 0.11 | -0.20 ± 0.60 | -4.15 (<.001) | 2.37 (0.125) |
|  | pericalcarine | 1.63 ± 0.12 | 1.63 ± 0.12 | -0.08 ± 0.99 | -1.02 (0.308) | 1.65 ± 0.12 | 1.62 ± 0.11 | -0.41 ± 0.85 | -6.15 (<.001) | 3.33 (0.069) |
|  | postcentral | 2.07 ± 0.11 | 2.05 ± 0.12 | -0.31 ± 0.70 | -5.47 (<.001) | 2.00 ± 0.12 | 1.97 ± 0.12 | -0.44 ± 0.54 | -10.14 (<.001) | 14.64 (<.001) |
|  | posteriorcingulate | 2.47 ± 0.14 | 2.46 ± 0.14 | -0.14 ± 0.69 | -2.50 (0.013) | 2.44 ± 0.15 | 2.41 ± 0.15 | -0.25 ± 0.59 | -5.32 (<.001) | 4.32 (0.039) |
|  | precentral | 2.47 ± 0.16 | 2.45 ± 0.15 | -0.28 ± 1.08 | -3.27 (0.001) | 2.49 ± 0.13 | 2.44 ± 0.14 | -0.49 ± 0.75 | -8.40 (<.001) | 11.14 (<.001) |
|  | precuneus | 2.38 ± 0.11 | 2.36 ± 0.12 | -0.25 ± 0.58 | -5.39 (<.001) | 2.29 ± 0.10 | 2.25 ± 0.11 | -0.40 ± 0.54 | -9.34 (<.001) | 1.05 (0.306) |
|  | rostralanteriorcingulate | 2.87 ± 0.20 | 2.89 ± 0.20 | 0.13 ± 0.76 | 2.14 (0.034) | 2.79 ± 0.21 | 2.81 ± 0.23 | 0.19 ± 0.81 | 2.90 (0.004) | 0.08 (0.773) |
|  | rostralmiddlefrontal | 2.32 ± 0.11 | 2.32 ± 0.12 | 0.06 ± 0.52 | 1.42 (0.159) | 2.26 ± 0.10 | 2.25 ± 0.11 | -0.19 ± 0.66 | -3.72 (<.001) | 4.01 (0.046) |
|  | superiorfrontal | 2.67 ± 0.13 | 2.66 ± 0.14 | -0.09 ± 0.53 | -2.09 (0.038) | 2.55 ± 0.11 | 2.52 ± 0.12 | -0.23 ± 0.65 | -4.51 (<.001) | 3.81 (0.052) |
|  | superiorparietal | 2.20 ± 0.13 | 2.18 ± 0.15 | -0.28 ± 0.62 | -5.65 (<.001) | 2.11 ± 0.12 | 2.08 ± 0.13 | -0.33 ± 0.58 | -7.17 (<.001) | 1.06 (0.303) |
|  | superiortemporal | 2.73 ± 0.13 | 2.70 ± 0.15 | -0.23 ± 0.56 | -5.19 (<.001) | 2.72 ± 0.14 | 2.67 ± 0.15 | -0.41 ± 0.52 | -9.97 (<.001) | 0.11 (0.737) |
|  | supramarginal | 2.51 ± 0.12 | 2.49 ± 0.15 | -0.18 ± 0.61 | -3.66 (<.001) | 2.44 ± 0.11 | 2.41 ± 0.12 | -0.38 ± 0.49 | -9.95 (<.001) | 7.47 (0.007) |
|  | temporalpole | 3.79 ± 0.31 | 3.74 ± 0.35 | -0.26 ± 0.91 | -3.58 (<.001) | 3.74 ± 0.26 | 3.69 ± 0.30 | -0.33 ± 0.75 | -5.50 (<.001) | 6.13 (0.014) |
|  | transversetemporal | 2.28 ± 0.23 | 2.27 ± 0.24 | -0.15 ± 1.35 | -1.43 (0.156) | 2.34 ± 0.22 | 2.25 ± 0.24 | -0.94 ± 1.16 | -10.23 (<.001) | 4.12 (0.043) |

Table S2: General linear Model assessing annual percentage changes (APC) of cortical thickness for the whole group. Values in italic survive multiple comparison using FDR correction. Values in Bold survive Bonferroni multiple comparison correction: Cortical thickness = p <.05 / 68 regions of interest.

|  |  | Region | Intercept | Age(TP1) | Sex | Education | Euler | Sample |
| --- | --- | --- | --- | --- | --- | --- | --- | --- |
| Whole | left | bankssts | 4.67 (0.032) | 5.60  (0.019) | 0.04 (0.834) | 2.00  (0.158) | 1.27 (0.261) | 0.43 (0.515) |
|  |  | caudalanteriorcingulate | 3.87  (0.05) | 4.82 (0.029) | 1.29 (0.256) | 1.20  (0.273) | 1.16 (0.281) | 1.74 (0.188) |
|  |  | caudalmiddlefrontal | 1.63 (0.203) | 2.04 (0.154) | 0.43 (0.513) | 0.12  (0.734) | 0.93 (0.335) | 1.99 (0.159) |
|  |  | cuneus | 0.81 (0.369) | 0.78 (0.377) | 1.18 (0.278) | 0.02  (0.888) | 0.00  (0.951) | 4.25  (0.04) |
|  |  | entorhinal | 7.71 (0.006) | 8.51 (0.004) | 0.01 (0.921) | 0.28  (0.594) | 2.97 (0.086) | 0.64 (0.424) |
|  |  | frontalpole | 0.1  (0.756) | 0.00  (0.948) | 2.5 (0.115) | 0.00  (0.96) | 2.09 (0.149) | 0.29 (0.593) |
|  |  | fusiform | 5.04 (0.025) | 5.53 (0.019) | 1.22 (0.27) | 0.02  (0.886) | 0.83 (0.362) | 1.38 (0.241) |
|  |  | inferiorparietal | 1.11 (0.293) | 1.45  (0.23) | 0.72 (0.395) | 0.8  (0.371) | 1.50  (0.222) | 3.06 (0.081) |
|  |  | inferiortemporal | 1.69 (0.195) | 2.14 (0.144) | 0.19 (0.663) | 0.12  (0.73) | 0.06 (0.807) | 0.01 (0.934) |
|  |  | insula | 1.15 (0.285) | 1.16 (0.282) | 0.57 (0.451) | 0.86  (0.354) | 0.04 (0.842) | 3.55 (0.061) |
|  |  | isthmuscingulate | 4.24  (0.04) | 5.25 (0.023) | 3.62 (0.058) | 1.45  (0.229) | 0.51 (0.475) | 0.11  (0.74) |
|  |  | lateraloccipital | 0.25  (0.62) | 0.16 (0.686) | 0.44 (0.507) | 0.00  (0.958) | 0.16 (0.694) | *9.32 (0.002)* |
|  |  | lateralorbitofrontal | 3.07 (0.081) | 2.46 (0.117) | 0.01 (0.942) | 4.81  (0.029) | 0.34 (0.563) | 0.75 (0.386) |
|  |  | lingual | 0.06 (0.809) | 0.00  (0.989) | 0.07 (0.795) | 0.45  (0.505) | 0.39 (0.531) | 4.09 (0.044) |
|  |  | MeanThickness | 1.83 (0.177) | 2.41 (0.121) | 0.00  (0.966) | 0.10  (0.756) | 0.94 (0.334) | 7.50  (0.007) |
|  |  | medialorbitofrontal | 2.85 (0.092) | 2.46 (0.118) | 0.01 (0.941) | 2.13  (0.146) | 1.87 (0.172) | *7.96 (0.005)* |
|  |  | middletemporal | 2.63 (0.106) | 3.99 (0.047) | 0.25 (0.62) | 0.68  (0.411) | 0.44 (0.507) | *8.53 (0.004)* |
|  |  | paracentral | 0.04 (0.838) | 0.02 (0.896) | 0.01 (0.914) | 0.23  (0.629) | 0.95  (0.330) | *8.97 (0.003)* |
|  |  | parahippocampal | 4.01 (0.046) | 3.34 (0.069) | 1.80 (0.181) | 0.32  (0.571) | 10.74 (0.001) | 0.04 (0.841) |
|  |  | parsopercularis | 0.19 (0.661) | 0.30  (0.583) | 0.16 (0.69) | 0.49  (0.482) | 0.06 (0.814) | 5.26 (0.023) |
|  |  | parsorbitalis | 0.69 (0.407) | 0.68 (0.411) | 1.98 (0.161) | 0.95  (0.331) | 1.46 (0.228) | 0.64 (0.425) |
|  |  | parstriangularis | 2.26 (0.134) | 2.19  (0.14) | 0.70 (0.403) | 0.11  (0.739) | 0.04 (0.834) | **13.67 (<.001)** |
|  |  | pericalcarine | 0.35 (0.554) | 0.01 (0.912) | 0.90 (0.344) | 0.06  (0.808) | 1.64 (0.201) | 0.46 (0.496) |
|  |  | postcentral | 2.53 (0.113) | 3.67 (0.056) | 0.85 (0.358) | 0.01  (0.913) | 2.44 (0.119) | 3.04 (0.082) |
|  |  | posteriorcingulate | 4.64 (0.032) | 4.38 (0.037) | 0.55 (0.457) | 0.06  (0.811) | 5.14 (0.024) | *5.87 (0.016)* |
|  |  | precentral | 1.25 (0.264) | 1.57 (0.212) | 0.41 (0.52) | 0.20  (0.655) | 5.14 (0.024) | *6.52 (0.011)* |
|  |  | precuneus | 0.67 (0.415) | 2.19  (0.140) | 0.08 (0.781) | 0.15  (0.699) | 1.73  (0.190) | 1.06 (0.304) |
|  |  | rostralanteriorcingulate | 5.12 (0.024) | 6.17 (0.014) | 0.10 (0.755) | 1.58  (0.21) | *9.72 (0.002)* | 4.84 (0.029) |
|  |  | rostralmiddlefrontal | 0.38 (0.536) | 0.23 (0.629) | 1.86 (0.174) | 0.63  (0.428) | 4.88 (0.028) | 3.72 (0.055) |
|  |  | superiorfrontal | 2.46 (0.118) | 3.07 (0.081) | 0.09 (0.760) | 0.74  (0.389) | 5.44  (0.020) | 2.49 (0.115) |
|  |  | superiorparietal | 0.20  (0.654) | 0.39 (0.532) | 0.06 (0.805) | 0.33  (0.567) | 2.49 (0.116) | 1.39  (0.240) |
|  |  | superiortemporal | 2.03 (0.155) | 4.32 (0.039) | 0.02 (0.899) | 0.12  (0.728) | 1.05 (0.305) | 0.06 (0.801) |
|  |  | supramarginal | 2.01 (0.157) | 2.49 (0.115) | 0.57 (0.451) | 0.56  (0.456) | 0.98 (0.322) | 5.15 (0.024) |
|  |  | temporalpole | 0.94 (0.332) | 1.67 (0.197) | 0.44 (0.507) | 0.03  (0.858) | 1.51 (0.219) | 0.88  (0.35) |
|  |  | transversetemporal | 4.74  (0.030) | 5.27 (0.022) | 0.08 (0.782) | 0.08  (0.772) | 0.02 (0.901) | *11.00*  *(0.001)* |
|  | right | bankssts | 0.00  (0.997) | 0.09 (0.762) | 0.00  (0.974) | 0.36  (0.548) | 1.34 (0.248) | 2.64 (0.105) |
|  |  | caudalanteriorcingulate | 2.08 (0.151) | 3.25 (0.072) | 0.09 (0.768) | 1.68  (0.196) | 6.44 (0.012) | 0.20  (0.658) |
|  |  | caudalmiddlefrontal | 0.24 (0.623) | 0.41 (0.525) | 0.10 (0.755) | 0.03  (0.854) | 0.48 (0.489) | *8.00*  *(0.005)* |
|  |  | cuneus | 5.35 (0.021) | 4.91 (0.027) | 0.56 (0.454) | 0.51  (0.478) | 0.01 (0.921) | *7.39 (0.007)* |
|  |  | entorhinal | 7.37 (0.007) | 7.42 (0.007) | 0.09 (0.769) | 2.29  (0.132) | 1.80  (0.181) | 1.37 (0.243) |
|  |  | frontalpole | 0.01 (0.931) | 0.04 (0.838) | 0.17 (0.677) | 1.17  (0.281) | 0.55  (0.460) | 1.15 (0.283) |
|  |  | fusiform | 6.45 (0.012) | 6.59 (0.011) | 0.85 (0.358) | 0.49  (0.486) | 1.41 (0.237) | 2.74 (0.099) |
|  |  | inferiorparietal | 0.00  (0.968) | 0.04 (0.835) | 0.20 (0.652) | 0.35  (0.552) | 3.21 (0.074) | 2.83 (0.093) |
|  |  | inferiortemporal | 11.55 (0.001) | **13.46 (<.001)** | 2.93 (0.088) | 0.01  (0.922) | 0.86 (0.353) | 0.80  (0.373) |
|  |  | insula | 5.94 (0.015) | 5.56 (0.019) | 2.51 (0.114) | 2.33  (0.128) | 0.58 (0.448) | *6.41 (0.012)* |
|  |  | isthmuscingulate | 0.04  (0.84) | 0.11 (0.743) | 4.10 (0.044) | 0.01  (0.916) | 0.04  (0.850) | 2.33 (0.128) |
|  |  | lateraloccipital | 0.04 (0.843) | 0.05 (0.824) | 0.00  (0.958) | 0.03  (0.856) | 2.25 (0.135) | *8.64 (0.004)* |
|  |  | lateralorbitofrontal | 3.65 (0.057) | 2.60  (0.108) | 0.52 (0.470) | 1.99  (0.159) | 0.09 (0.765) | 4.33 (0.038) |
|  |  | lingual | 0.34 (0.558) | 0.63 (0.427) | 1.08  (0.300) | 0.01  (0.924) | 1.09 (0.298) | 2.10  (0.148) |
|  |  | MeanThickness | 4.35 (0.038) | 4.95 (0.027) | 0.44 (0.508) | 0.95  (0.331) | 0.32 (0.572) | 8.85 (0.003) |
|  |  | medialorbitofrontal | 5.99 (0.015) | 6.06 (0.014) | 0.07 (0.795) | 0.10  (0.749) | 0.20  (0.658) | 2.63 (0.106) |
|  |  | middletemporal | 4.03 (0.046) | 4.43 (0.036) | 3.21 (0.074) | 0.19  (0.66) | 0.09 (0.768) | 0.36 (0.551) |
|  |  | paracentral | 1.53 (0.217) | 2.12 (0.146) | 0.41 (0.523) | 0.24  (0.621) | 0.04 (0.842) | 2.66 (0.104) |
|  |  | parahippocampal | 7.65 (0.006) | 10.14 (0.002) | 0.04 (0.842) | 0.00  (0.980) | 1.89 (0.171) | 1.20  (0.274) |
|  |  | parsopercularis | 3.36 (0.068) | 1.35 (0.246) | 0.00  (0.986) | 4.39  (0.037) | 10.00  (0.002) | **21.43 (<.001)** |
|  |  | parsorbitalis | 1.32 (0.252) | 0.70  (0.403) | 0.08 (0.782) | 0.04  (0.850) | 2.43  (0.120) | 5.22 (0.023) |
|  |  | parstriangularis | 0.67 (0.414) | 0.13  (0.720) | 0.06 (0.812) | 1.27  (0.261) | 1.37 (0.242) | **16.54 (<.001)** |
|  |  | pericalcarine | 4.00  (0.046) | 3.50  (0.062) | 0.12 (0.734) | 0.00  (0.976) | 0.08 (0.775) | *8.33 (0.004)* |
|  |  | postcentral | 0.05 (0.823) | 0.35 (0.557) | 0.04 (0.839) | 0.20  (0.651) | 0.67 (0.413) | 3.30  (0.070) |
|  |  | posteriorcingulate | 2.18 (0.141) | 2.67 (0.103) | 0.44 (0.507) | 0.04  (0.845) | 0.00  (1.00) | 1.62 (0.204) |
|  |  | precentral | 1.12 (0.292) | 0.85 (0.358) | 2.57 (0.110) | 2.67  (0.103) | 1.27 (0.261) | 4.19 (0.042) |
|  |  | precuneus | 1.21 (0.272) | 2.72  (0.100) | 0.88 (0.349) | 0.00  (0.998) | 0.00  (0.959) | 3.48 (0.063) |
|  |  | rostralanteriorcingulate | 0.04 (0.834) | 0.02 (0.892) | 0.42 (0.517) | 0.00  (0.968) | 0.23 (0.629) | 0.69 (0.407) |
|  |  | rostralmiddlefrontal | 6.15 (0.014) | 6.43 (0.012) | 0.29 (0.591) | 0.00  (0.994) | 0.73 (0.393) | *7.99 (0.005)* |
|  |  | superiorfrontal | 3.16 (0.076) | 3.75 (0.054) | 0.04 (0.849) | 1.34  (0.248) | 1.53 (0.217) | *6.09 (0.014)* |
|  |  | superiorparietal | 0.13 (0.723) | 0.75 (0.388) | 1.40 (0.238) | 0.73  (0.393) | 0.16  (0.690) | 0.05 (0.819) |
|  |  | superiortemporal | 1.92 (0.166) | 2.81 (0.095) | 0.68 (0.412) | 0.46  (0.498) | 2.62 (0.106) | *10.66 (0.001)* |
|  |  | supramarginal | 5.63 (0.018) | 5.96 (0.015) | 0.00  (0.973) | 0.32  (0.572) | 2.91 (0.089) | *10.82 (0.001)* |
|  |  | temporalpole | 2.23 (0.136) | 2.81 (0.094) | 0.00  (0.966) | 0.09  (0.768) | 0.88 (0.348) | 0.72 (0.397) |
|  |  | transversetemporal | 1.88 (0.172) | 2.02 (0.157) | 1.15 (0.284) | 0.51  (0.474) | 0.31 (0.581) | **20.47 (<.001)** |

Table S3: General linear Model assessing annual percentage changes (APC) for the two samples separately. Values in Bold survive multiple comparison correction: Cortical thickness = p <.05 / 68 regions of interest.

|  |  | Region | Intercept | Age(TP1) | Sex | Education | Euler |
| --- | --- | --- | --- | --- | --- | --- | --- |
| 1000BRAINS | left | bankssts | 1.81 (0.181) | 1.97 (0.162) | 0.03 (0.856) | 2.29  (0.133) | 1.26 (0.263) |
|  |  | caudalanteriorcingulate | 0.27 (0.601) | 0.47 (0.493) | 0.55 (0.461) | 1.11  (0.294) | 0.52 (0.471) |
|  |  | caudalmiddlefrontal | 0.41 (0.524) | 0.54 (0.465) | 0.11 (0.742) | 0.38  (0.541) | 0.29 (0.592) |
|  |  | cuneus | 0.11 (0.741) | 0.05 (0.815) | 0.19 (0.661) | 0.03  (0.874) | 0.11 (0.737) |
|  |  | entorhinal | 3.34  (0.070) | 3.9  (0.050) | 0.02 (0.883) | 0.31  (0.581) | 1.17  (0.280) |
|  |  | frontalpole | 0.10  (0.757) | 0.27 (0.602) | 1.70  (0.194) | 0.13  (0.721) | 3.75 (0.055) |
|  |  | fusiform | 1.96 (0.163) | 2.17 (0.143) | 1.56 (0.214) | 0.10  (0.754) | 0.95 (0.332) |
|  |  | inferiorparietal | 0.30  (0.583) | 0.53 (0.469) | 0.87 (0.354) | 0.07  (0.786) | 1.01 (0.317) |
|  |  | inferiortemporal | 0.35 (0.557) | 0.57 (0.452) | 0.73 (0.395) | 0.27  (0.601) | 0.01 (0.906) |
|  |  | insula | 1.62 (0.205) | 1.58  (0.210) | 1.13 (0.289) | 0.24  (0.624) | 0.01 (0.917) |
|  |  | isthmuscingulate | 1.24 (0.268) | 1.32 (0.253) | 3.57 (0.061) | 1.27  (0.262) | 0.09 (0.767) |
|  |  | lateraloccipital | 0.09 (0.762) | 0.01 (0.928) | 0.70  (0.404) | 0.80  (0.371) | 0.16  (0.690) |
|  |  | lateralorbitofrontal | 1.69 (0.196) | 1.27 (0.262) | 1.97 (0.162) | 2.88  (0.092) | 1.20  (0.276) |
|  |  | lingual | 0.16 (0.693) | 0.47 (0.492) | 0.00  (0.991) | 1.79  (0.183) | 0.49 (0.486) |
|  |  | MeanThickness | 0.43 (0.515) | 0.76 (0.383) | 0.07 (0.797) | 0.02  (0.899) | 0.46  (0.500) |
|  |  | medialorbitofrontal | 1.42 (0.235) | 1.10  (0.295) | 0.00  (0.969) | 4.77  (0.03) | 4.46 (0.036) |
|  |  | middletemporal | 0.38 (0.537) | 0.76 (0.384) | 0.20  (0.656) | 0.09  (0.769) | 0.30  (0.587) |
|  |  | paracentral | 0.06 (0.805) | 0.07 (0.798) | 0.15  (0.700) | 0.09  (0.771) | 0.30  (0.586) |
|  |  | parahippocampal | 0.00  (0.959) | 0.01 (0.943) | 0.56 (0.456) | 0.09  (0.770) | 6.61 (0.011) |
|  |  | parsopercularis | 0.12 (0.731) | 0.18 (0.671) | 0.02 (0.896) | 0.41  (0.525) | 0.52 (0.473) |
|  |  | parsorbitalis | 0.76 (0.386) | 0.68  (0.410) | 0.07 (0.789) | 0.20  (0.657) | 1.20  (0.274) |
|  |  | parstriangularis | 2.03 (0.156) | 2.19 (0.141) | 0.19  (0.660) | 0.52  (0.471) | 0.05 (0.825) |
|  |  | pericalcarine | 0.03 (0.862) | 0.15 (0.704) | 2.85 (0.094) | 0.89  (0.347) | 2.63 (0.107) |
|  |  | postcentral | 2.84 (0.094) | 3.82 (0.052) | 0.79 (0.375) | 0.02  (0.879) | 0.96 (0.329) |
|  |  | posteriorcingulate | 0.00  (0.980) | 0.00  (0.998) | 1.03 (0.311) | 0.06  (0.803) | 6.62 (0.011) |
|  |  | precentral | 1.09 (0.299) | 1.43 (0.234) | 0.11 (0.746) | 0.32  (0.571) | 2.28 (0.133) |
|  |  | precuneus | 0.48 (0.487) | 1.45  (0.230) | 0.02 (0.902) | 0.12  (0.733) | 2.60  (0.109) |
|  |  | rostralanteriorcingulate | 8.97 (0.003) | 8.73 (0.004) | 1.25 (0.265) | 7.39  (0.007) | **13.31 (<.001)** |
|  |  | rostralmiddlefrontal | 0.11 (0.737) | 0.03 (0.858) | 1.73 (0.191) | 1.29  (0.258) | 5.14 (0.025) |
|  |  | superiorfrontal | 0.44 (0.506) | 0.66 (0.417) | 0.20  (0.656) | 0.05  (0.821) | 5.5  (0.02) |
|  |  | superiorparietal | 0.86 (0.354) | 1.41 (0.237) | 1.79 (0.183) | 0.14  (0.709) | 1.17 (0.282) |
|  |  | superiortemporal | 0.01 (0.914) | 0.27 (0.605) | 0.01 (0.914) | 0.07  (0.795) | 0.60  (0.438) |
|  |  | supramarginal | 0.94 (0.333) | 1.24 (0.267) | 0.5 0  (0.481) | 0.20  (0.657) | 0.47 (0.492) |
|  |  | temporalpole | 0.24 (0.622) | 0.53  (0.47) | 1.02 (0.314) | 0.00  (0.949) | 2.47 (0.118) |
|  |  | transversetemporal | 3.24 (0.074) | 3.57 (0.061) | 0.02 (0.881) | 0.31  (0.581) | 0.26  (0.61) |
|  | right | bankssts | 0.03 (0.867) | 0.02 (0.893) | 0.42 (0.516) | 0.00  (0.958) | 2.11 (0.149) |
|  |  | caudalanteriorcingulate | 1.61 (0.207) | 2.03 (0.156) | 0.01 (0.923) | 1.58  (0.211) | 3.63 (0.059) |
|  |  | caudalmiddlefrontal | 0.10  (0.755) | 0.09 (0.759) | 0.01 (0.933) | 0.48  (0.489) | 0.22 (0.642) |
|  |  | cuneus | 2.21 (0.139) | 1.49 (0.224) | 0.01 (0.941) | 2.60  (0.109) | 0.06 (0.814) |
|  |  | entorhinal | 6.58 (0.011) | 7.15 (0.008) | 0.01 (0.943) | 0.78  (0.377) | 0.62 (0.433) |
|  |  | frontalpole | 0.20  (0.659) | 0.15 (0.695) | 0.00  (0.981) | 0.25  (0.616) | 0.57  (0.45) |
|  |  | fusiform | 3.67 (0.057) | 3.74 (0.055) | 0.46  (0.500) | 0.38  (0.537) | 0.74 (0.391) |
|  |  | inferiorparietal | 0.23 (0.634) | 0.29 (0.594) | 0.07 (0.791) | 1.28  (0.259) | 1.82 (0.179) |
|  |  | inferiortemporal | 5.6  (0.019) | 6.32 (0.013) | 3.13 (0.079) | 0.01  (0.935) | 0.6 0  (0.441) |
|  |  | insula | 0.28 (0.598) | 0.20  (0.654) | 1.14 (0.287) | 1.19  (0.277) | 0.24 (0.623) |
|  |  | isthmuscingulate | 0.20  (0.659) | 0.15 (0.701) | 6.27 (0.013) | 0.17  (0.678) | 0.13 (0.716) |
|  |  | lateraloccipital | 0.67 (0.413) | 0.65 (0.421) | 0.14 (0.707) | 0.03  (0.855) | 0.84 (0.362) |
|  |  | lateralorbitofrontal | 0.92 (0.339) | 0.36 (0.548) | 0.99  (0.320) | 3.23  (0.074) | 0.02 (0.891) |
|  |  | lingual | 0.71 (0.402) | 1.19 (0.278) | 0.05 (0.822) | 0.00  (0.980) | 1.83 (0.178) |
|  |  | MeanThickness | 2.72 (0.101) | 2.72 (0.101) | 1.00  (0.319) | 1.99  (0.160) | 0.15 (0.701) |
|  |  | medialorbitofrontal | 0.25  (0.620) | 0.34 (0.562) | 0.15 (0.698) | 1.27  (0.262) | 0.34 (0.561) |
|  |  | middletemporal | 0.74 (0.391) | 0.64 (0.426) | 5.38 (0.022) | 0.13  (0.718) | 0.01 (0.916) |
|  |  | paracentral | 0.75 (0.388) | 1.50  (0.223) | 4.48 (0.036) | 0.00  (0.965) | 0.14 (0.713) |
|  |  | parahippocampal | 2.08 (0.151) | 3.29 (0.072) | 0.63 (0.429) | 0.19  (0.667) | 1.15 (0.286) |
|  |  | parsopercularis | 3.78 (0.054) | 1.87 (0.173) | 0.29 (0.589) | 5.07  (0.026) | 6.69 (0.011) |
|  |  | parsorbitalis | 1.22 (0.272) | 0.53 (0.467) | 0.25 (0.619) | 0.13  (0.721) | 3.86 (0.051) |
|  |  | parstriangularis | 0.17 (0.677) | 0.02 (0.902) | 0.41 (0.524) | 2.58  (0.111) | 2.77 (0.098) |
|  |  | pericalcarine | 1.53 (0.218) | 0.95  (0.33) | 0.81 (0.369) | 0.43  (0.513) | 0.43 (0.514) |
|  |  | postcentral | 0.37 (0.544) | 0.14 (0.714) | 0.11 (0.746) | 0.10  (0.752) | 0.41 (0.525) |
|  |  | posteriorcingulate | 2.48 (0.118) | 2.39 (0.124) | 0.02 (0.875) | 1.74  (0.189) | 0  (0.998) |
|  |  | precentral | 0.18 (0.669) | 0.05 (0.815) | 3.16 (0.077) | 1.78  (0.185) | 1.18 (0.278) |
|  |  | precuneus | 1.44 (0.233) | 2.82 (0.095) | 0.84  (0.360) | 0.08  (0.782) | 0.24 (0.628) |
|  |  | rostralanteriorcingulate | 0.24 (0.622) | 0.16 (0.694) | 0.07 (0.787) | 0.19  (0.66) | 0.41 (0.524) |
|  |  | rostralmiddlefrontal | 6.54 (0.012) | 6.31 (0.013) | 0.11 (0.746) | 0.47  (0.496) | 1.77 (0.186) |
|  |  | superiorfrontal | 3.31 (0.071) | 3.71 (0.056) | 0.00  (0.980) | 0.09  (0.761) | 0.56 (0.456) |
|  |  | superiorparietal | 0.11 (0.746) | 0.48  (0.490) | 2.77 (0.098) | 1.19  (0.277) | 0.20  (0.653) |
|  |  | superiortemporal | 0.80  (0.373) | 0.82 (0.367) | 2.14 (0.145) | 0.05  (0.830) | 3.19 (0.076) |
|  |  | supramarginal | 8.44 (0.004) | 7.63 (0.006) | 1.29 (0.258) | 1.93  (0.167) | 1.75 (0.188) |
|  |  | temporalpole | 0.41 (0.524) | 0.68 (0.412) | 0.02 (0.886) | 0.11  (0.742) | 0.78 (0.377) |
|  |  | transversetemporal | 0.58 (0.448) | 0.57 (0.451) | 0.47 (0.494) | 0.35  (0.557) | 0.05 (0.818) |
| LHAB | left | bankssts | 2.66 (0.105) | 3.59  (0.060) | 0.47 (0.494) | 0.04  (0.837) | 0.44 (0.508) |
|  |  | caudalanteriorcingulate | 5.56  (0.020) | 5.82 (0.017) | 0.60  (0.440) | 0.18  (0.673) | 0.23 (0.631) |
|  |  | caudalmiddlefrontal | 0.92 (0.339) | 1.30  (0.256) | 0.21  (0.650) | 0.07  (0.793) | 2.12 (0.147) |
|  |  | cuneus | 1.29 (0.258) | 1.15 (0.286) | 1.22 (0.271) | 0.12  (0.726) | 0.11 (0.739) |
|  |  | entorhinal | 4.24 (0.041) | 4.14 (0.044) | 0.17 (0.679) | 0.04  (0.840) | 5.08 (0.026) |
|  |  | frontalpole | 0.89 (0.348) | 0.75 (0.388) | 0.94 (0.334) | 0.21  (0.651) | 0.79 (0.375) |
|  |  | fusiform | 3.25 (0.073) | 3.92 (0.049) | 0.09 (0.767) | 0.43  (0.512) | 0.04 (0.836) |
|  |  | inferiorparietal | 0.57  (0.45) | 0.89 (0.348) | 0.05 (0.819) | 1.17  (0.281) | 0.31  (0.58) |
|  |  | inferiortemporal | 2.12 (0.147) | 2.09 (0.151) | 0.06 (0.804) | 1.43  (0.234) | 0.00  (0.955) |
|  |  | insula | 0.01 (0.915) | 0.11 (0.739) | 0.01 (0.939) | 0.76  (0.386) | 0.25 (0.616) |
|  |  | isthmuscingulate | 3.62 (0.059) | 5.3  (0.023) | 0.43 (0.514) | 0.24  (0.622) | 0.36 (0.549) |
|  |  | lateraloccipital | 0.39 (0.532) | 0.23 (0.633) | 0.00  (0.965) | 1.66  (0.199) | 0.08 (0.779) |
|  |  | lateralorbitofrontal | 1.75 (0.188) | 1.13 (0.289) | 2.77 (0.098) | 1.95  (0.165) | 4.43 (0.037) |
|  |  | lingual | 1.12 (0.292) | 0.78 (0.378) | 0.32  (0.57) | 0.69  (0.407) | 0.20  (0.652) |
|  |  | MeanThickness | 1.02 (0.315) | 1.55 (0.215) | 0.02 (0.894) | 0.37  (0.545) | 0.96 (0.329) |
|  |  | medialorbitofrontal | 0.62 (0.431) | 0.72 (0.397) | 0.02 (0.902) | 0.10  (0.757) | 3.48 (0.064) |
|  |  | middletemporal | 5.14 (0.025) | 4.76 (0.031) | 0.03  (0.87) | 1.14  (0.288) | 0.02 (0.881) |
|  |  | paracentral | 0.10  (0.758) | 0.04  (0.84) | 0.05 (0.819) | 0.12  (0.727) | 1.09 (0.299) |
|  |  | parahippocampal | **12.28 (0.001)** | 9.52 (0.002) | 0.96 (0.328) | 2.34  (0.128) | 7.91 (0.006) |
|  |  | parsopercularis | 0.01  (0.940) | 0.04 (0.851) | 0.83 (0.364) | 0.06  (0.804) | 3.25 (0.073) |
|  |  | parsorbitalis | 0.21 (0.647) | 0.22 (0.641) | 4.83  (0.030) | 0.98  (0.323) | 0.87 (0.352) |
|  |  | parstriangularis | 0.99 (0.321) | 0.34 (0.559) | 0.78 (0.379) | 0.15  (0.698) | 0.12 (0.733) |
|  |  | pericalcarine | 0.71 (0.401) | 0.54 (0.465) | 0.2  (0.654) | 0.49  (0.487) | 0.02 (0.896) |
|  |  | postcentral | 0.00  (0.954) | 0.22 (0.641) | 0.05 (0.822) | 0.00  (0.978) | 0.49 (0.485) |
|  |  | posteriorcingulate | 9.00  (0.003) | 10.40 (0.002) | 0.01 (0.936) | 0.65  (0.423) | 0.00  (0.983) |
|  |  | precentral | 0.02 (0.876) | 0.15 (0.701) | 0.45 (0.503) | 0.00  (0.967) | 2.57 (0.111) |
|  |  | precuneus | 0.13 (0.718) | 0.62 (0.432) | 0.10  (0.752) | 0.03  (0.866) | 0.34 (0.563) |
|  |  | rostralanteriorcingulate | 0.23 (0.631) | 0.40  (0.526) | 0.33 (0.566) | 0.85  (0.357) | 0.01 (0.912) |
|  |  | rostralmiddlefrontal | 0.07 (0.799) | 0.20  (0.658) | 0.44 (0.508) | 0.00  (0.983) | 0.20  (0.657) |
|  |  | superiorfrontal | 1.45  (0.230) | 2.06 (0.154) | 0.34 (0.564) | 1.02  (0.315) | 1.79 (0.183) |
|  |  | superiorparietal | 0.40  (0.527) | 0.24 (0.624) | 1.09 (0.298) | 0.15  (0.701) | 0.42  (0.520) |
|  |  | superiortemporal | 4.23 (0.041) | 6.46 (0.012) | 0.21  (0.650) | 0.08  (0.783) | 0.00  (0.992) |
|  |  | supramarginal | 0.55  (0.460) | 1.07 (0.302) | 0.12 (0.732) | 0.36  (0.552) | 0.47 (0.496) |
|  |  | temporalpole | 1.07 (0.303) | 1.19 (0.276) | 0.10  (0.756) | 0.05  (0.831) | 3.52 (0.063) |
|  |  | transversetemporal | 0.41 (0.522) | 1.10  (0.297) | 0.38 (0.536) | 0.19  (0.661) | 0.91  (0.340) |
|  | right | bankssts | 0.02 (0.893) | 0.87 (0.351) | 0.68 (0.412) | 1.18  (0.280) | 1.10  (0.296) |
|  |  | caudalanteriorcingulate | 0.53 (0.467) | 1.38 (0.242) | 0.22 (0.638) | 0.26  (0.610) | 3.7  (0.056) |
|  |  | caudalmiddlefrontal | 0.00  (0.953) | 0.23  (0.630) | 0.38 (0.538) | 1.2  (0.275) | 1.06 (0.306) |
|  |  | cuneus | 3.79 (0.053) | 3.89  (0.050) | 1.16 (0.283) | 0.78  (0.380) | 0.33 (0.566) |
|  |  | entorhinal | 2.02 (0.158) | 1.45 (0.231) | 0.09 (0.761) | 1.45  (0.231) | 1.63 (0.204) |
|  |  | frontalpole | 0.50  (0.479) | 0.56 (0.455) | 0.29 (0.592) | 1.20  (0.275) | 0.07 (0.794) |
|  |  | fusiform | 2.05 (0.154) | 2.54 (0.113) | 0.35 (0.553) | 0.09  (0.759) | 0.85 (0.358) |
|  |  | inferiorparietal | 0.64 (0.424) | 0.11 (0.741) | 0.97 (0.326) | 0.29  (0.594) | 1.27 (0.262) |
|  |  | inferiortemporal | 5.64 (0.019) | 7.26 (0.008) | 0.41 (0.523) | 0.00  (0.973) | 0.06 (0.803) |
|  |  | insula | 7.98 (0.005) | 8.88 (0.003) | 0.99  (0.32) | 1.23  (0.270) | 0.06 (0.811) |
|  |  | isthmuscingulate | 0.04 (0.847) | 0.05 (0.832) | 0.04 (0.841) | 0.12  (0.729) | 0.02 (0.887) |
|  |  | lateraloccipital | 3.24 (0.074) | 2.32  (0.130) | 0.11 (0.738) | 0.00  (0.985) | 0.84  (0.36) |
|  |  | lateralorbitofrontal | 2.43 (0.121) | 2.9  (0.090) | 0.02 (0.895) | 0.00  (0.995) | 0.07 (0.786) |
|  |  | lingual | 0.25 (0.617) | 0.13 (0.718) | 3.99 (0.048) | 0.00  (0.977) | 0.17 (0.678) |
|  |  | MeanThickness | 1.02 (0.315) | 2.11 (0.149) | 0.01 (0.927) | 0.01  (0.921) | 0.56 (0.455) |
|  |  | medialorbitofrontal | 7.56 (0.007) | 7.47 (0.007) | 0.02 (0.892) | 0.39  (0.533) | 0.63 (0.429) |
|  |  | middletemporal | 5.04 (0.026) | 6.33 (0.013) | 0.00  (0.995) | 0.11  (0.743) | 0.09 (0.761) |
|  |  | paracentral | 0.16 (0.694) | 0.23 (0.635) | 2.58  (0.110) | 0.73  (0.395) | 0.34 (0.559) |
|  |  | parahippocampal | 6.97 (0.009) | 7.51 (0.007) | 0.42 (0.518) | 0.33  (0.568) | 0.57 (0.452) |
|  |  | parsopercularis | 0.02 (0.882) | 0.00  (0.954) | 0.40  (0.528) | 0.09  (0.764) | 1.1  (0.295) |
|  |  | parsorbitalis | 0.10  (0.752) | 0.4  (0.527) | 0.58 (0.449) | 0.41  (0.521) | 0.75 (0.389) |
|  |  | parstriangularis | 0.15 (0.703) | 0.68 (0.412) | 1.06 (0.306) | 0.02  (0.889) | 0.70  (0.405) |
|  |  | pericalcarine | 3.61 (0.059) | 3.38 (0.068) | 0.28 (0.596) | 0.77  (0.383) | 0.40  (0.529) |
|  |  | postcentral | 0.96  (0.330) | 1.86 (0.174) | 0.01  (0.93) | 0.10  (0.756) | 1.24 (0.268) |
|  |  | posteriorcingulate | 0.00  (0.978) | 0.27 (0.604) | 0.68 (0.411) | 2.22  (0.139) | 0.14 (0.704) |
|  |  | precentral | 1.31 (0.253) | 1.95 (0.165) | 0.04  (0.850) | 0.92  (0.338) | 0.31 (0.578) |
|  |  | precuneus | 0.01 (0.926) | 0.24 (0.625) | 0.13 (0.719) | 0.08  (0.778) | 0.56 (0.457) |
|  |  | rostralanteriorcingulate | 0.02 (0.881) | 0.06  (0.810) | 0.41 (0.522) | 0.20  (0.658) | 0.05 (0.825) |
|  |  | rostralmiddlefrontal | 0.78 (0.377) | 1.34 (0.249) | 0.98 (0.324) | 0.45  (0.503) | 0.62 (0.432) |
|  |  | superiorfrontal | 0.21 (0.645) | 0.54 (0.464) | 0.03 (0.874) | 1.95  (0.165) | 2.06 (0.154) |
|  |  | superiorparietal | 0.01 (0.938) | 0.13 (0.715) | 0.00  (0.990) | 0.00  (0.985) | 0.08 (0.779) |
|  |  | superiortemporal | 0.72 (0.399) | 2.63 (0.107) | 0.14 (0.712) | 1.63  (0.204) | 0.02 (0.875) |
|  |  | supramarginal | 0.03 (0.854) | 0.27 (0.603) | 1.62 (0.206) | 0.95  (0.332) | 0.32 (0.571) |
|  |  | temporalpole | 2.42 (0.122) | 2.87 (0.092) | 0.02 (0.902) | 0.98  (0.325) | 0.04 (0.836) |
|  |  | transversetemporal | 0.55 (0.459) | 1.78 (0.185) | 0.72 (0.397) | 0.17  (0.683) | 0.67 (0.414) |

Table S4: Relation between annual percentage changes (APC) in cortical thickness with APC in cognitive performance, calculated using general linear models for the two samples, corrected for age, sex, education and data quality (Euler number). None of the results survived multiple comparison correction (FDR or Bonferroni).

|  |  | Processing Speed | | Concept Shifting | | Verbal Fluency | | Reasoning | |
| --- | --- | --- | --- | --- | --- | --- | --- | --- | --- |
|  |  | 1000B | LHAB | 1000B | LHAB | 1000B | LHAB | 1000B | LHAB |
| Left | bankssts | 2.01 (0.158) | 6.26 (0.013) | 0.04 (0.838) | 0.09 (0.761) | 0.00 (0.953) | 0.39 (0.531) | 0.54 (0.465) | 0.01 (0.934) |
|  | caudalanteriorcingulate | 0.06 (0.810) | 2.73 (0.100) | 0.38 (0.541) | 4.29 (0.040) | 3.54 (0.062) | 0.06 (0.811) | 0.28 (0.598) | 1.32 (0.252) |
|  | caudalmiddlefrontal | 0.66 (0.417) | 2.49 (0.117) | 0.06 (0.807) | 1.97 (0.163) | 0.03 (0.857) | 0.07 (0.794) | 2.11 (0.149) | 2.45 (0.120) |
|  | cuneus | 1.00 (0.318) | 0.06 (0.811) | 1.93 (0.167) | 0.38 (0.537) | 1.34 (0.249) | 5.60 (0.019) | 3.86 (0.052) | 1.01 (0.317) |
|  | entorhinal | 1.43 (0.234) | 0.14 (0.706) | 0.38 (0.538) | 0.19 (0.660) | 1.66 (0.199) | 0.07 (0.798) | 0.01 (0.925) | 3.34 (0.070) |
|  | fusiform | 1.19 (0.277) | 1.00 (0.318) | 0.00 (0.997) | 0.03 (0.852) | 2.96 (0.087) | 1.50 (0.223) | 3.05 (0.083) | 0.00 (0.946) |
|  | inferiorparietal | 0.13 (0.720) | 10.7 (0.001) | 0.00 (0.945) | 0.13 (0.721) | 3.75 (0.055) | 0.15 (0.698) | 0.02 (0.895) | 0.29 (0.592) |
|  | inferiortemporal | 0.16 (0.691) | 3.05 (0.083) | 2.14 (0.146) | 0.86 (0.354) | 0.01 (0.937) | 0.24 (0.624) | 2.98 (0.087) | 0.05 (0.822) |
|  | isthmuscingulate | 4.71 (0.032) | 3.88 (0.051) | 0.26 (0.609) | 0.08 (0.775) | 0.17 (0.679) | 1.00 (0.320) | 0.84 (0.360) | 2.10 (0.150) |
|  | lateraloccipital | 0.24 (0.624) | 1.89 (0.171) | 1.72 (0.192) | 0.01 (0.933) | 0.98 (0.323) | 0.00 (0.996) | 0.04 (0.849) | 0.27 (0.605) |
|  | lateralorbitofrontal | 4.77 (0.030) | 2.48 (0.117) | 0.13 (0.714) | 0.20 (0.652) | 0.34 (0.558) | 0.02 (0.901) | 6.77 (0.010) | 0.72 (0.396) |
|  | lingual | 0.20 (0.654) | 0.13 (0.714) | 0.45 (0.503) | 0.25 (0.615) | 0.03 (0.869) | 1.96 (0.164) | 0.35 (0.557) | 0.53 (0.467) |
|  | medialorbitofrontal | 0.07 (0.791) | 0.00 (0.968) | 0.00 (0.972) | 2.67 (0.104) | 0.13 (0.716) | 0.00 (0.970) | 1.98 (0.162) | 1.14 (0.288) |
|  | middletemporal | 1.38 (0.243) | 4.74 (0.031) | 0.52 (0.471) | 0.45 (0.502) | 0.01 (0.923) | 0.88 (0.349) | 0.01 (0.918) | 0.08 (0.784) |
|  | parahippocampal | 0.17 (0.680) | 1.11 (0.294) | 0.09 (0.766) | 0.30 (0.582) | 0.06 (0.805) | 0.23 (0.631) | 2.46 (0.119) | 1.71 (0.193) |
|  | paracentral | 0.37 (0.546) | 1.67 (0.198) | 0.14 (0.708) | 1.6 0(0.208) | 0.07 (0.788) | 0.62 (0.433) | 2.83 (0.095) | 0.04 (0.848) |
|  | parsopercularis | 3.31 (0.071) | 5.19 (0.024) | 0.95 (0.332) | 0.14 (0.712) | 0.12 (0.731) | 0.09 (0.771) | 4.3 (0.040) | 0.22 (0.643) |
|  | parsorbitalis | 0.64 (0.427) | 0.74 (0.392) | 0.72 (0.398) | 0.25 (0.618) | 0.01 (0.919) | 3.85 (0.052) | 0.01 (0.938) | 0.02 (0.875) |
|  | parstriangularis | 0.98 (0.323) | 3.37 (0.068) | 0.25 (0.620) | 0.10 (0.753) | 1.11 (0.293) | 0.00 (0.961) | 4.86 (0.029) | 0.58 (0.449) |
|  | pericalcarine | 0.63 (0.427) | 0.47 (0.494) | 0.65 (0.421) | 3.49 (0.064) | 0.21 (0.644) | 0.49 (0.487) | 0.00 (0.981) | 0.11 (0.746) |
|  | postcentral | 1.24 (0.268) | 13.49 (0.000) | 0.28 (0.597) | 0.05 (0.823) | 0.74 (0.391) | 0.09 (0.767) | 0.08 (0.773) | 1.86 (0.175) |
|  | posteriorcingulate | 0.01 (0.908) | 0.01 (0.932) | 0.00 (0.965) | 0.55 (0.460) | 0.12 (0.731) | 3.61 (0.059) | 0.05 (0.828) | 0.01 (0.918) |
|  | precentral | 0.04 (0.845) | 2.08 (0.151) | 0.24 (0.624) | 0.63 (0.427) | 0.44 (0.508) | 0.66 (0.419) | 2.61 (0.109) | 1.51 (0.221) |
|  | precuneus | 0.11 (0.740) | 4.98 (0.027) | 0.03 (0.859) | 0.00 (0.984) | 0.09 (0.770) | 0.16 (0.685) | 0.59 (0.443) | 2.69 (0.103) |
|  | rostralanteriorcingulate | 0.21 (0.651) | 0.38 (0.539) | 0.59 (0.443) | 0.00 (0.947) | 6.44 (0.012) | 0.23 (0.630) | 0.10 (0.757) | 0.01 (0.931) |
|  | rostralmiddlefrontal | 0.00 (0.986) | 3.13 (0.079) | 0.07 (0.793) | 0.40 (0.526) | 0.37 (0.545) | 0.24 (0.627) | 1.76 (0.187) | 0.27 (0.605) |
|  | superiorfrontal | 0.06 (0.808) | 0.94 (0.334) | 3.74 (0.055) | 0.01 (0.915) | 0.28 (0.595) | 0.16 (0.692) | 0.02 (0.876) | 0.84 (0.362) |
|  | superiorparietal | 1.8 (0.182) | 8.3 (0.005) | 0.13 (0.715) | 0.00 (0.975) | 0.73 (0.393) | 1.24 (0.268) | 0.09 (0.764) | 2.07 (0.152) |
|  | superiortemporal | 0.62 (0.434) | 5.61 (0.019) | 0.10 (0.748) | 0.18 (0.669) | 0.16 (0.693) | 0.38 (0.537) | 4.34 (0.039) | 0.88 (0.351) |
|  | supramarginal | 1.51 (0.221) | 11.14 (0.001) | 1.82 (0.179) | 0.00 (0.971) | 0.50 (0.482) | 0.04 (0.840) | 2.38 (0.125) | 0.03 (0.857) |
|  | frontalpole | 1.90 (0.170) | 0.00 (0.972) | 1.12 (0.292) | 0.68 (0.410) | 0.29 (0.590) | 0.02 (0.877) | 0.67 (0.413) | 0.01 (0.916) |
|  | temporalpole | 0.00 (0.987) | 0.02 (0.892) | 0.09 (0.770) | 2.93 (0.089) | 0.14 (0.708) | 0.03 (0.869) | 0.00 (0.954) | 0.34 (0.562) |
|  | transversetemporal | 0.96 (0.330) | 2.49 (0.117) | 1.97 (0.162) | 0.00 (0.995) | 0.68 (0.410) | 0.28 (0.597) | 2.16 (0.144) | 0.15 (0.699) |
|  | insula | 0.33 (0.565) | 5.29 (0.023) | 0.73 (0.395) | 0.55 (0.460) | 0.24 (0.628) | 0.00 (0.984) | 0.14 (0.714) | 1.85 (0.176) |
|  | MeanThickness | 0.21 (0.651) | 5.45 (0.021) | 1.27 (0.263) | 0.31 (0.581) | 0.26 (0.609) | 0.00 (0.997) | 2.40 (0.124) | 1.03 (0.311) |
| RIGHT | bankssts | 1.55 (0.215) | 1.05 (0.307) | 0.35 (0.553) | 1.19 (0.276) | 0.27 (0.607) | 0.32 (0.573) | 0.72 (0.396) | 3.39 (0.068) |
|  | caudalanteriorcingulate | 1.47 (0.228) | 0.56 (0.455) | 2.30 (0.132) | 1.38 (0.242) | 2.86 (0.093) | 0.71 (0.402) | 0.76 (0.386) | 0.21 (0.651) |
|  | caudalmiddlefrontal | 0.13 (0.724) | 0.59 (0.442) | 0.27 (0.604) | 0.00 (0.946) | 0.00 (0.951) | 0.00 (0.957) | 0.73 (0.396) | 3.99 (0.048) |
|  | cuneus | 0.03 (0.872) | 0.4 (0.527) | 0.02 (0.876) | 1.11 (0.295) | 7.97 (0.005) | 4.69 (0.032) | 0.32 (0.571) | 0.23 (0.632) |
|  | entorhinal | 0.16 (0.691) | 9.20 (0.003) | 0.11 (0.742) | 1.23 (0.269) | 1.24 (0.267) | 0.20 (0.654) | 0.41 (0.522) | 0.61 (0.436) |
|  | fusiform | 0.32 (0.573) | 0.93 (0.336) | 0.03 (0.869) | 0.52 (0.474) | 4.02 (0.047) | 0.74 (0.392) | 1.37 (0.243) | 1.06 (0.305) |
|  | inferiorparietal | 0.16 (0.689) | 0.43 (0.511) | 1.01 (0.317) | 0.06 (0.800) | 1.31 (0.254) | 0.04 (0.845) | 0.41 (0.522) | 0.17 (0.68) |
|  | inferiortemporal | 4.58 (0.034) | 3.06 (0.083) | 0.15 (0.700) | 0.5 (0.481) | 0.12 (0.730) | 0.04 (0.848) | 0.00 (0.998) | 0.76 (0.384) |
|  | isthmuscingulate | 1.16 (0.282) | 0.01 (0.927) | 0.84 (0.362) | 0.20 (0.657) | 3.18 (0.076) | 0.03 (0.872) | 0.46 (0.498) | 1.99 (0.161) |
|  | lateraloccipital | 1.25 (0.265) | 0.27 (0.607) | 1.85 (0.175) | 0.38 (0.540) | 2.56 (0.112) | 1.39 (0.241) | 0.57 (0.450) | 0.15 (0.704) |
|  | lateralorbitofrontal | 2.15 (0.145) | 1.19 (0.277) | 0.00 (0.980) | 0.07 (0.795) | 0.2 (0.658) | 1.00 (0.318) | 0.05 (0.827) | 2.59 (0.110) |
|  | lingual | 0.10 (0.748) | 0.18 (0.671) | 0.37 (0.546) | 3.66 (0.058) | 0.77 (0.381) | 3.70 (0.056) | 0.38 (0.540) | 0.00 (0.969) |
|  | medialorbitofrontal | 1.83 (0.178) | 3.94 (0.049) | 0.66 (0.418) | 0.01 (0.903) | 0.94 (0.335) | 0.23 (0.629) | 1.76 (0.187) | 3.00 (0.086) |
|  | middletemporal | 1.16 (0.284) | 0.98 (0.324) | 1.98 (0.161) | 0.36 (0.549) | 1.95 (0.165) | 0.00 (0.967) | 0.90 (0.346) | 1.70 (0.194) |
|  | parahippocampal | 1.24 (0.266) | 2.47 (0.118) | 0.01 (0.929) | 0.96 (0.329) | 0.97 (0.327) | 0.71 (0.402) | 0.01 (0.929) | 0.37 (0.543) |
|  | paracentral | 0.80 (0.374) | 0.4 (0.527) | 0.00 (0.949) | 0.15 (0.701) | 0.00 (0.949) | 0.10 (0.748) | 0.85 (0.36) | 0.38 (0.54) |
|  | parsopercularis | 1.06 (0.305) | 0.63 (0.429) | 0.11 (0.745) | 0.28 (0.597) | 0.21 (0.649) | 2.52 (0.115) | 0.03 (0.873) | 1.79 (0.182) |
|  | parsorbitalis | 0.88 (0.350) | 0.1 (0.757) | 0.61 (0.438) | 0.52 (0.472) | 1.63 (0.204) | 0.01 (0.927) | 0.03 (0.860) | 1.61 (0.206) |
|  | parstriangularis | 0.08 (0.780) | 0.67 (0.413) | 5.69 (0.018) | 1.94 (0.166) | 7.35 (0.008) | 0.00 (1.00) | 1.50 (0.223) | 2.96 (0.088) |
|  | pericalcarine | 0.38 (0.537) | 0.07 (0.797) | 0.58 (0.449) | 0.56 (0.455) | 5.19 (0.024) | 4.28 (0.040) | 0.04 (0.839) | 0.42 (0.520) |
|  | postcentral | 4.45 (0.037) | 0.08 (0.781) | 0.01 (0.920) | 0.50 (0.479) | 0.05 (0.820) | 0.30 (0.583) | 0.01 (0.921) | 2.15 (0.145) |
|  | posteriorcingulate | 0.40 (0.530) | 4.94 (0.028) | 0.63 (0.428) | 1.17 (0.282) | 1.26 (0.264) | 4.20 (0.042) | 1.45 (0.231) | 4.01 (0.047) |
|  | precentral | 0.34 (0.559) | 0.12 (0.729) | 0.65 (0.423) | 0.03 (0.860) | 1.10 (0.296) | 0.16 (0.694) | 0.30 (0.582) | 1.77 (0.185) |
|  | precuneus | 0.98 (0.323) | 0.14 (0.713) | 0.70 (0.403) | 0.01 (0.905) | 0.16 (0.687) | 0.35 (0.555) | 2.26 (0.135) | 1.64 (0.202) |
|  | rostralanteriorcingulate | 2.25 (0.136) | 1.88 (0.172) | 0.02 (0.890) | 0.94 (0.334) | 1.02 (0.315) | 1.70 (0.195) | 0.05 (0.828) | 0.44 (0.509) |
|  | rostralmiddlefrontal | 3.21 (0.075) | 2.69 (0.103) | 0.03 (0.860) | 2.26 (0.135) | 2.08 (0.151) | 1.08 (0.300) | 0.04 (0.849) | 2.80 (0.097) |
|  | superiorfrontal | 6.95 (0.009) | 1.54 (0.216) | 0.52 (0.472) | 0.49 (0.486) | 0.49 (0.486) | 0.08 (0.772) | 0.60 (0.440) | 3.14 (0.078) |
|  | superiorparietal | 0.06 (0.814) | 0.04 (0.841) | 0.02 (0.893) | 0.23 (0.630) | 0.11 (0.746) | 0.12 (0.731) | 0.24 (0.623) | 0.65 (0.420) |
|  | superiortemporal | 0.11 (0.74) | 3.70 (0.056) | 0.02 (0.893) | 0.00 (0.952) | 0.58 (0.449) | 0.19 (0.663) | 0.11 (0.743) | 0.77 (0.381) |
|  | supramarginal | 2.81 (0.096) | 1.05 (0.307) | 0.02 (0.892) | 0.72 (0.397) | 0.00 (0.970) | 0.92 (0.340) | 0.05 (0.816) | 2.31 (0.130) |
|  | frontalpole | 0.04 (0.847) | 1.23 (0.269) | 1.69 (0.196) | 0.18 (0.670) | 0.58 (0.448) | 0.29 (0.591) | 0.39 (0.534) | 7.41 (0.007) |
|  | temporalpole | 0.70 (0.403) | 3.55 (0.062) | 0.79 (0.375) | 3.17 (0.077) | 0.71 (0.401) | 0.19 (0.665) | 0.02 (0.899) | 0.34 (0.563) |
|  | transversetemporal | 0.00 (0.976) | 0.23 (0.632) | 1.3 (0.257) | 2.60 (0.109) | 0.38 (0.539) | 0.02 (0.881) | 0.16 (0.692) | 0.15 (0.700) |
|  | insula | 1.49 (0.224) | 0.95 (0.331) | 0.20 (0.657) | 0.36 (0.547) | 2.01 (0.159) | 0.03 (0.854) | 0.73 (0.395) | 0.20 (0.658) |
|  | MeanThickness | 1.55 (0.215) | 2.63 (0.107) | 0.03 (0.864) | 0.00 (0.971) | 0.45 (0.505) | 0.41 (0.522) | 0.49 (0.484) | 3.5 (0.063) |
